# Interpreting small treatment differences from quality of life data in cancer trials: an alternative measure of treatment benefit and effect size for the EORTC-QLQ-C30

**DOI:** 10.1186/s12955-015-0374-6

**Published:** 2015-11-14

**Authors:** Iftekhar Khan, Zahid Bashir, Martin Forster

**Affiliations:** Department of Applied Health Research, University College London, 1-19 Torrington Place, London, WC1E 7HB UK; School of Cancer Sciences, University of Birmingham, Edgbaston, Birmingham, B15 2TT UK; University College London, University College Hospital, 235 Euston Rd, London, NW1 2BU UK

**Keywords:** EORTC-QLQ-C30, Lung cancer, Quality of life, Beta binomial, Treatment effect size, MD: Mean Differences, ORs: Odds Ratios

## Abstract

**Background:**

The EORTC-QLQ-C30 is a widely used health related quality of life (HRQoL) questionnaire in lung cancer patients. Small HRQoL treatment effects are often reported as mean differences (MDs) between treatments, which are rarely justified or understood by patients and clinicians. An alternative approach using odds ratios (OR) for reporting effects is proposed. This may offer advantages including facilitating alignment between patient and clinician understanding of HRQoL effects.

**Methods:**

Data from six CRUK sponsored randomized controlled lung cancer trials (2 small cell and 4 in non-small cell, in 2909 patients) were used to HRQoL effects. Results from Beta-Binomial (BB) standard mixed effects were compared. Preferences for ORs vs MDs were determined and Time to Deterioration (TD) was also compared.

**Results:**

HRQoL effects using ORs offered coherent interpretations: MDs >0 resulted in ORs >1 and vice versa; effect sizes were classified as ‘Trivial’ if the OR was between 1 ± 0.05 (i.e. 0.95 to 1.05); ‘Small’: for 1 ± 0.1; ‘Medium’: 1 ± 0.2 and ‘Large’: OR <0.8 or >1.20. Small HRQoL effects on the MD scale may translate to important treatment differences on the OR scale: for example, a worsening in symptoms (MD) by 2.6 points (*p* = 0.1314) would be a 17 % deterioration (*p* < 0.0001) with an OR. Hence important differences may be missed with MD; conversely, small ORs are unlikely to yield large MDs because methods based on OR model skewed data well. Initial evidence also suggests oncologists prefer ORs over MDs since interpretation is similar to hazard ratios.

**Conclusion:**

Reporting HRQoL benefits as MDs can be misleading. Estimates of HRQoL treatment effects in terms of ORs are preferred over MDs. Future analysis of QLQ-C30 and other HRQoL measures should consider reporting HRQoL treatment effects as ORs.

**Electronic supplementary material:**

The online version of this article (doi:10.1186/s12955-015-0374-6) contains supplementary material, which is available to authorized users.

## Background

Health related quality of life (HRQoL) is an important endpoint in cancer trials for several reasons. First, where effect sizes are small, HRQoL can ‘add value’ to expensive cancer treatments. Secondly, considerable time is spent completing instruments for the purpose of estimating the impact of treatments on HRQoL. Therefore, such efforts should result in HRQoL effects that are meaningful and interpretable, especially where HRQoL is a primary or co-primary endpoint [1]. Thirdly, some anti-cancer treatments exhibit serious side-effects, despite improvements in overall survival (OS); HRQoL is also reported to be a predictor of survival in lung cancer patients [2], the leading cause of death among cancers [3]. It would be important to understand for example, how survival differs between patients with ‘poor’ baseline HRQoL, compared to those with ‘Good’ HRQoL. Finally, HRQoL outcomes are often required for cost-effectiveness analyses and drug reimbursement [4, 5]. Therefore, understanding and interpreting HRQoL data is crucial in evaluating cancer treatments.

The EORTC-QLQ-C30 (QLQ-C30) is a widely used cancer specific instrument [6]. The instrument has 30 questions from which 15 domains (sub scales) are determined, consisting of 5 ‘function’ scales, 8 ‘symptom’ scales, a global quality of life (QL) scale and a finance scale (FI). For QL and function domains, high scores indicate better HRQoL. For symptom domains (and FI), low scores indicate better HRQoL.

Treatment effects from the QLQ-C30 are often reported as mean differences (MDs) [7], despite scores having heavily skewed distributions with ceiling effects (many patients with scores of 0 or 100) and censored data due to progressive disease, death or failure to complete questionnaires. The interpretation of HRQoL MDs can be more complicated than survival endpoints. Consequently, alternative measures of treatment effect have been proposed.

Maringwa suggests a minimally important ‘*difference over time*’ as a measure of effect [8]. The area under the curve (AUC) can be difficult to interpret, although useful for reducing multiple observations to a single value [9]. However, if HRQoL is measured at a few time points (e.g. baseline and month 12), the AUC will have limited value. Moreover, the interpretation of the effect can become tricky (e.g. for HRQoL scores of 100 at each of 0, 1 and 2 months, the AUC score is but the original HRQoL scale is 0 to 100).

Categorizing scores: e.g. improvements in symptoms from ‘moderate’ or ‘severe’ (67–100 points at baseline) to ‘non’ or ‘little’ (0 to 33 points) was proposed by Langendjik [10]. Reck and Norman [11, 12] suggested ‘noted’ changes in HRQoL occur when a ‘shift’ of greater than half of the baseline standard deviation is observed). Time to HRQoL deterioration (TD) has been suggested (Anota) [13]. However different definitions of ‘deterioration’ lead to different conclusions and median TD may not be estimable (e.g. few events) and further complicated by non-proportional hazards (PH). Interpretation of effects with TD using HRs is however similar to ORs. Reporting a ‘Trend’ is also a way of describing HRQoL over time (Schaake) [14], although difficult to interpret (e.g. how much ‘more trend’ is there for experimental vs. control?).

The above measures of HRQoL effects can be difficult to interpret for patients and clinicians. The mean is often the statistic of choice to define treatment effect sizes for HRQoL endpoints in most of these measures.

One commonly reported clinically relevant effect size proposed by Osoba and King [6, 15, 16] is ≥10 points MD (on any domain), a value used as a benchmark by researchers to determine whether HRQoL benefits exist [7]. Some researchers interpret a 10 point improvement as a *difference between treatments*, while others as a 10 point *change (improvement) from baseline* (Hirsh) [6, 17], which is not always possible. For example, if a patient scores 8 points (or 92 points) at baseline, a reduction (or increase) of 10 points is not possible. Moreover, ‘important’ treatment differences need not be the same for symptom as functional scales. A *worsening* of 5 points in a symptom scale may be more important than a 10 point *improvement* in a functional scale.

For HRQoL endpoints, the magnitude of effect sizes are often considered to be clinically relevant if a difference of 10 points is observed, regardless of whether HRQoL is a primary or secondary outcome. Such requirements are not expected of other secondary clinical endpoints in cancer trials (e.g. time to progression (TTP)). One reason may be that secondary endpoints are not powered or there is a clinical rationale that the secondary outcome cannot be expected to yield effects similar to primary endpoints. In a similar vein, effect sizes should not be expected to be uniform across HRQoL domains for demonstrating treatment benefit because some smaller effect sizes (e.g. < 10 points) may be important. In this research we attempt to show that some small effect sizes on a MD scale might be dismissed as clinically irrelevant but remain important on a relative scale.

Little attention has been given to smaller HRQoL effects (MDs) which are often glossed over unless a ‘statistically significant’ *p*-value is reported alongside. Small MDs tend to be perceived as offering limited HRQoL benefit but can mask important improvements, particularly when data are analysed using an alternative scale (e.g. OR scale). This presents a challenge for setting thresholds for defining clinically relevant HRQoL effect sizes. Moreover, ORs can facilitate an interpretation of effects similar to hazard ratios (HR), familiar to many oncologists (OR are interpreted in a similar way to HRs).

Therefore, in this article after presenting baseline characteristics, we offer effect size categories based on the OR and describe example situations of the relationship between ORs and MDs. We discuss aspects of statistical significance of small effects in the context of ORs and MDs and compare preferences between ORs vs MDs from several clinicians; Finally, we compare ORs and MDs with time a to deterioration (TD) approach (TD ≥5 points) following Anota [13].

## Methods

### Data

HRQoL data from six randomized controlled trials (RCT) conducted by the CRUK & UCL CTC were analayzed [9, 18–22]. These were selected because they comprised of all patient level QLQ-C30 data available in the CTC database from RCTs in lung cancer which had been published.

(i) ‘TOPICAL’: A phase III trial in NSCLC patients unfit for chemotherapy comparing erlotinib with placebo [18]; *N* = 670 patients.
(ii) ‘SOCCAR’: A phase II trial comparing concurrent vs. sequential chemotherapy in NSCLC patients [19]; *N* = 130.
(iii) ‘Study 10’: A phase II trial comparing Gemcitabine/Carboplatin versus Cisplatin/Etoposide in patients with small cell lung cancer (SCLC) [20]; N = 241.
(iv) ‘Study 11’: A phase III trial comparing Gemcitabine/Carboplatin versus Mitomycin/Ifosfamide /Cisplatin in patients with stage IIIB or IV NSCLC [9]; *N* = 422
(v) ‘Study 12’: A phase III trial comparing Thalidomide combined with chemotherapy versus chemotherapy alone in SCLC patients [21]; *N* = 724
(vi) Study 14: A phase III trial comparing Thalidomide/Gemcitabine/Carboplatin versus Gemcitabine/Carboplatin alone in NSCLC patients [22]; *N* = 722

### Assessments

Data were collected during clinic visits and questionnaires returned by patients during follow up; QLQ-C30 was assessed at several time points including baseline, pre and post chemotherapy and at monthly intervals for at least 24 months or until disease progression.

### Statistical analysis

Patient level HRQoL scores for each of the 15 domain scores were analysed using a a repeated measures [21, 22] analysis for reporting MDs and a more novel Beta Binomial (BB) model in a mixed model framework [23] for reporting ORs. For the BB model, responses were transformed to a (0,1) scale using the transformation [23] Y-a/b-a, where a and b are the minimum and maximum possible scores and Y the observed response. For example, a score of 80 is transformed as 80- 0/(100-a) = 80/100 = 0.8. Dichotomization is not required for a BB model to generate ORs.

The BB model has been used in a variety of applications [23–25]. Its advantages over standard (linear) models in terms of statistical properties are widely reported [25, 26]. The BB is also flexible because it models scores at the extreme ends of the scale (e.g. many patients scoring 0 or 100), a common feature of QLQ-C30 scores, using zero–one inflated model [25, 26]. MDs were classified similar to those described by Cocks [7]; ‘Trivial’ (0–3 points), ‘Small’ (3–10 points), ‘Modest’/ ‘Medium’ (10–15 points) and ‘Large’ (>15 points). Similarly, ORs were classified as 1 ± 0.05 (‘Trivial’), 1 ± 0.1 (‘Small’), 1 ± 0.2 (‘Medium’) and <0.8 or >1.2 (‘Large’). Time to Deterioration (TD) was determined using the first time where scores reduced/increased by ≥ 5 points. Patients without deterioration were censored. A Kaplan-Meier and Cox proportional hazards (PH) analysis was carried out.

A pilot survey was carried out to determine preliminary evidence of whether clinicians and/or patients preferred ORs or MDs for expressing treatment effects. Three items, physical function (PF), Pain (PA) and cognitive function (CF) from the 15 domains were randomly selected and presented to each of five clinicians and their patients (where possible). Patients/clinicians were asked to state preferences for ORs or MDs (Additional file 1). Lower/High scores express preferences for ORs; scores close to 5 express indifference.

## Results

### Demographics and baseline characteristics

The median age was 64 years (range 27–86 years) with oldest patients in the TOPICAL trial (median age 77); 61 % were male; 67 % were ECOG (0–1), 24 % ECOG 2 and 9 % ECOG 3 (Table 1); less than half were stage IIIa-IIIb (47 %) [9, 18–22]. Most QLQ-C30 responses were >90 % complete at baseline (Additional file 2: Table S1) with the exception of study 10 (about 60 % complete). More than 50 % of data were available for at least 5 time points.

**Table 1:** Summary of baseline characteristics for each trial

	TOPICAL (*N* = 670)	SOCCAR (*N* = 130)	Study 10 (*N* = 241)	Study 11 (*N* = 422)	Study 12 (*N* = 724)	Study 14 (*N* = 722)
Age (Median, range)	77 (72–82)	62 (39–75)	62 (27–81)	62 (34–81)	65 (38–86)	62 (33–84)
Gender:						
Male	409 (61 %)	79 (61 %)	136 (56 %)	296 (70 %)	412 (57 %)	465 (64 %)
Female	261 (39 %)	51 (39 %)	105 (43 %)	126 (30 %)	312 (43 %)	257 (36 %)
ECOG: 0–1	106 (16 %)	130 (100 %)	164 (68 %)	365 (86 %)	529 (73 %)	648 (90 %)
2	372 (56 %)	0	60 (25 %)	48 (11 %)	153 (21 %)	74 (10 %)
3	192 (29 %)	0	17 (7 %)	9 (21 %)	42 ( 6 %)	0
Stage: IIIa-IIIb	234 (35 %)	130 (100 %)	103 (43 %)	200 (47 %)	368^a^(51 %)	322 (45 %)
IV	436 (65 %)	0	138 (57 %)	222 (53 %	356^a^(49 %)	400 (55 %)

^a^limited disease *n* = 368, extensive disease *n* = 356

Eastern Co-operative Oncology Group (ECOG) status:

0: Fully active, able to carry on all pre-disease performance without restriction

1 Restricted in physically strenuous activity but ambulatory and able to carry out work of a light or sedentary nature, e.g., light house work, office work

2 Ambulatory and capable of all self-care but unable to carry out any work activities. Up and about more than 50 % of waking hours

3 Capable of only limited self-care, confined to bed or chair more than 50 % of waking hours

4 Completely disabled. Cannot carry on any selfcare. Totally confined to bed or chair

5 Dead

### Distribution of QLQ-C30

**Fig. 1:**
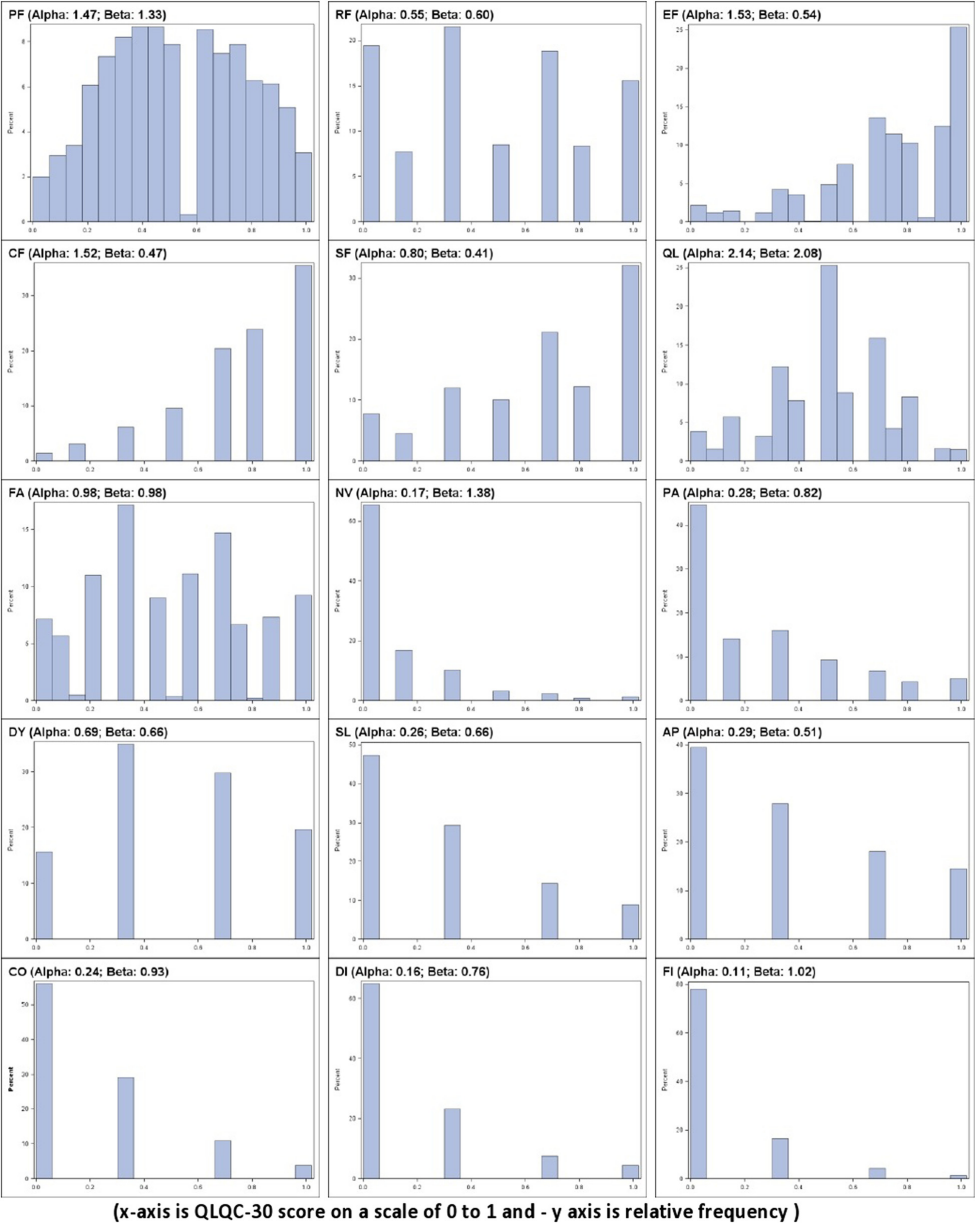
Distribution of QLQ-C30 responses: TOPICAL (x-axis is QLQ-C30 score on a scale of 0 to 1 and - y axis is relative frequency)

Most (>85 %) QLQ-C30 responses were very skewed (Fig. 1 & Additional file 2: Figure S1). For TOPICAL, 14/15 (93 %) of scores had alpha or beta values (special values associated with a BB distribution relating to the mean and variance) <1; Kolmogorov-Smirnov tests rejected normality (*p*-value <0.001). Therefore, using the mean as a measure of HRQoL benefit and consequently MDs is not considered a suitable reporting metric for HRQoL scores. Statistical analysis should be conducted according to the underlying (true) distribution of the data. The distribution of QLQ-C30 scores from the six trials were not normally distributed in most (≥85 %) of cases.

### Relationship between MDs and ORs

Few 4/90 (4 %) HRQoL treatment effects (MDs) were ‘Large’ (>15 points) or ‘Medium’ (10–15 points); 27/90 (30 %) were ‘Small’ (3–10 points) and 59/90 (66 %) ‘Trivial’ (0–3 points) MDs; For ORs, 22/90 (24 %) were ‘Large’ (effects > 20 %) or ‘Medium’ (effects between 10 % to 20 %) with the rest being ‘Small’ or ‘Trivial (10 % and 5 % respectively). ORs were therefore more than seven times more likely to detect larger differences which can yield up to 20 % improvements in HRQoL ([0.24/0.76]/[0.04/0.96]) compared with MDs (Tables 2 and 3).

**Table 2:** Mean differences compared with odds ratios

		Odds ratio				Mean Difference (MD)			
Study	QLQ-C30	Odds Ratio	Lower 95 % CI	Upper 95 % CI	*P*-value	Difference	Lower 95 % CI	Upper 95 % CI	*P*-value
TOPICAL	QL	0.99840	0.92173	1.08145	0.4687	0.6433	−1.4409	2.7274	0.5450
(*N* = 670)	PF	1.10396	1.01803	1.19714	0.0168	3.2075	1.2060	5.2090	0.0017
	SF	1.00312	0.90378	1.11339	0.4932	0.9528	−2.0474	3.9529	0.5334
	RF	1.07206	0.96858	1.18660	0.1790	2.2751	−0.8554	5.4055	0.1542
	EF	1.13356	1.02036	1.25931	0.0196	2.3352	−0.2060	4.8763	0.0717
	CF	1.14062	1.03104	1.26184	0.0107	3.6824	1.5497	5.8152	0.0007
	FA	0.97062	0.88076	1.06965	0.5472	0.4928	−2.0393	3.0249	0.7027
	NV	1.03299	0.90587	1.17796	0.6279	2.0823	0.2119	3.9527	0.0291
	PA	0.84858	0.75492	0.95386	0.0060	−4.1552	−6.7318	−1.5786	0.0016
	DY	0.90265	0.83349	0.97755	0.0118	−6.9802	−9.8067	−4.1538	<.0001
	SL	1.06142	0.96736	1.16463	0.2078	−0.5849	−3.4526	2.2829	0.6892
	AP	1.14413	1.04285	1.25524	0.0044	7.7375	4.3808	11.0943	<.0001
	CO	0.94016	0.85740	1.03090	0.1892	−9.3181	−11.9276	−6.7085	<.0001
	DI	1.11676	0.99977	1.24744	0.0505	15.0773	12.5221	17.6324	<.0001
	FI	1.06304	0.94126	1.20057	0.3245	−3.9678	−5.8688	−2.0668	<.0001
SOCCAR	QL	1.05911	0.94600	1.18576	0.3186	−0.3363	−3.2927	2.6201	0.8234
(*N* = 130)	PF	1.00632	0.88085	1.14967	0.5260	−0.7855	−3.5393	1.9684	0.5758
	SF	1.07805	0.94331	1.23204	0.2696	−0.1569	−3.8385	3.5246	0.9333
	RF	0.92530	0.81065	1.05617	0.2497	−1.2639	−5.1956	2.6679	0.5283
	EF	0.86964	0.74753	1.01171	0.0704	−0.4552	−3.7208	2.8104	0.7845
	CF	1.21469	1.04469	1.41237	0.0115	2.8249	0.2230	5.4268	0.0334
	FA	1.09003	0.96258	1.23436	0.1740	−3.2001	−6.5586	0.1585	0.0618
	NV	0.96687	0.81577	1.14595	0.4073	1.1136	−1.6169	3.8441	0.4237
	PA	0.93405	0.81386	1.07199	0.3313	−3.3510	−6.7785	0.07654	0.0553
	DY	0.90277	0.81740	0.99707	0.0436	−2.0557	−5.5569	1.4456	0.2495
	SL	1.07573	0.95694	1.20927	0.2211	−0.8873	−4.4778	2.7033	0.6278
	AP	0.94817	0.83543	1.07613	0.4095	−0.2265	−4.0527	3.5997	0.9075
	CO	1.10100	0.95927	1.26368	0.1709	−0.8742	−4.4752	2.7267	0.6339
	DI	0.84506	0.68652	1.04021	0.1121	−0.4002	−2.8583	2.0580	0.7494
	FI	1.10633	0.94859	1.29029	0.1977	1.1951	−2.1001	4.4903	0.4768
Study 10	QL	0.94126	0.84476	1.05135	0.3912	−1.6241	−4.1816	1.8922	0.6124
(*N* = 241)	PF	1.00563	0.89071	1.13537	0.9276	2.8207	−1.9594	7.6007	0.2467
	SF	1.00006	0.88002	1.13647	0.9993	4.0507	−0.9736	9.0750	0.1139
	RF	1.28860	1.08877	1.50121	0.0084	13.0540	0.3211	25.7870	0.0445
	EF	1.07878	0.93850	1.24003	0.2156	−2.8502	−7.4563	1.7558	0.2248
	CF	1.14441	0.96173	1.29133	0.1494	5.6888	2.0359	9.3417	0.0023
	FA	0.87034	0.77408	0.97858	0.0203	−2.0359	−6.1599	2.0882	0.3327
	NV	1.10180	0.94137	1.28957	0.2269	0.7394	−2.9807	4.4594	0.6965
	PA	0.81452	0.70091	0.94655	0.0075	−0.9057	−5.0882	3.2768	0.6708
	DY	0.90282	0.82439	0.98871	0.0275	−6.8389	−11.3035	−2.3743	0.0027
	SL	0.95514	0.85447	1.06767	0.4188	−0.3213	−5.8080	5.1655	0.9085
	AP	1.08583	0.95447	1.23526	0.2104	2.2718	−2.6561	7.1997	0.3657
	CO	0.86569	0.75412	0.99376	0.0405	−2.6788	−7.7010	2.3433	0.2953
	DI	0.92437	0.77487	1.10271	0.3818	−3.4211	−6.4418	−0.4004	0.0265
	FI	1.06566	0.82875	1.27688	0.3624	2.1354	−2.1141	6.5714	0.3252
Study 11	QL	0.95236	0.89286	1.09135	0.5552	−2.3613	−5.1511	2.9822	0.7334
(*N* = 422)	PF	0.93089	0.84991	1.01958	0.1229	−2.7725	−6.0805	0.5356	0.1003
	SF	0.96121	0.87398	1.05714	0.4147	−3.3172	−6.9318	0.2974	0.0720
	RF	1.08853	0.94125	1.23477	0.3324	−7.1287	−14.3603	0.1028	0.0533
	EF	0.97549	0.88043	1.08081	0.6350	−4.8538	−8.0029	−1.7047	0.0026
	CF	0.95256	0.85588	1.06016	0.3731	−0.6141	−3.1705	1.9422	0.6374
	FA	1.10834	1.00731	1.21950	0.0349	3.3375	0.2209	6.4541	0.0359
	NV	1.29384	1.15215	1.42351	<0.0001	5.4863	3.3832	7.5895	<.0001
	PA	1.00011	0.89283	1.12028	0.9985	0.9084	−1.9043	3.7210	0.5264
	DY	1.04262	0.96229	1.12965	0.3073	−1.6110	−5.1973	1.9754	0.3783
	SL	1.02938	0.94433	1.12209	0.5102	−4.0055	−7.6480	−0.3629	0.0312
	AP	1.04393	0.94403	1.15440	0.4019	4.6020	0.7884	8.4157	0.0181
	CO	1.06104	0.95671	1.17675	0.1117	2.6096	−0.7817	6.0008	0.1314
	DI	1.13002	0.95487	1.33729	0.1547	0.9181	−0.9858	2.8219	0.3442
	FI	1.08816	0.92995	1.22408	0.4498	1.9684	−2.0451	5.6714	0.5022
Study 12	QL	0.96311	0.89103	1.04421	0.5034	−0.8545	−2.8925	1.19985	0.3214
(*N* = 724)	PF	1.00156	0.93624	1.07143	0.9639	−0.01443	−2.9124	2.8835	0.9922
	SF	0.93058	0.87602	0.98855	0.0196	0.2535	−2.2932	2.8002	0.8453
	RF	0.94478	0.89925	0.99755	0.0536	−6.3209	−11.8670	−0.7747	0.0255
	EF	0.96366	0.90173	1.02985	0.2747	−4.0689	−6.3917	−1.7461	0.0006
	CF	0.96082	0.89968	1.02612	0.1334	−1.2498	−3.1386	0.6391	0.1946
	FA	0.99963	0.94240	1.06033	0.4901	−0.6901	−2.8719	1.4917	0.5352
	NV	0.95818	0.88480	1.03765	0.2933	5.4863	3.3832	7.5895	<.0001
	PA	0.96961	0.89701	1.04809	0.4370	0.9084	−1.9043	3.7210	0.5264
	DY	1.03922	0.98989	1.09102	0.1210	3.9991	1.3876	6.6105	0.0027
	SL	0.90162	0.84630	0.96055	0.0014	−15.5470	−18.0563	−13.0378	<.0001
	AP	1.06787	0.99510	1.14597	0.0682	−2.2811	−4.6592	0.09705	0.0601
	CO	1.16848	1.09944	1.24186	<.0001	2.6096	−0.7817	6.0008	0.1314
	DI	1.05255	0.95709	1.15754	0.2909	−2.3379	−3.8004	−0.8754	0.0017
	FI	1.01842	0.92606	1.12000	0.7064	1.6197	−2.5375	5.7768	0.4445
Study 14	QL	0.96684	0.91236	1.05549	0.2123	−1.3897	−3.2450	0.9808	0.0983
(*N* = 722)	PF	0.95134	0.89346	1.01298	0.1193	−1.7843	−3.6934	0.1248	0.0670
	SF	1.01050	0.94612	1.07926	0.7557	0.8731	−1.1044	2.8507	0.3867
	RF	0.92540	0.89452	0.99358	0.0412	−6.0800	−10.1493	−2.0107	0.0034
	EF	0.89881	0.83696	0.96523	0.0034	−1.9677	−3.5103	−0.4251	0.0124
	CF	1.04520	0.96868	1.12776	0.2543	−0.07931	−1.4565	1.2978	0.9101
	FA	1.03864	0.97432	1.10720	0.2450	1.1338	−0.5280	2.7955	0.1811
	NV	0.92440	0.84363	1.01291	0.0920	−1.7769	−3.0100	−0.5437	0.0048
	PA	1.07278	0.98702	1.16600	0.0984	−0.5521	−2.2252	1.1210	0.5176
	DY	1.02074	0.96756	1.07683	0.4520	1.3292	−0.5879	3.2464	0.1741
	SL	0.96340	0.89862	1.03286	0.2937	−8.0830	−10.1365	−6.0295	<.0001
	AP	0.98802	0.91442	1.06755	0.7602	−3.8491	−5.8767	−1.8216	0.0002
	CO	1.07744	1.00746	1.15228	0.0295	10.9195	8.8799	12.9590	<.0001
	DI	1.05388	0.95077	1.16817	0.3177	−1.6529	−2.8431	−0.4628	0.0065
	FI	0.99605	0.89553	1.10785	0.9418	2.3020	−2.4081	7.0122	0.3375

*Key*: 5 functional scales: *PF* physical function, *RF* role function, *EF* emotional function, *CF* cognitive function, and *SF* social functioning; 9 ‘symptom’ scales: *FA* fatigue, *NV* nausea & vomiting, *PA* pain, *DY* dyspnoea, *IN* insomnia, *AL* appetite loss, *CO* constipation, *DI* diarrhoea, *FI* financial problems; and *QL* a global health status score

Positive differences on the functional scale are improvements in quality of life with the experimental arm

Positive differences on the symptom scale suggests a worsening in quality of life with the experimental arm

**Table 3:** Magnitude of effect sizes

	All	Stat. sig^a^
Mean Difference	*n* (%)	*n* (%)
Trivial: 0–3 points	59 (66 %)	6 (19 %)
Small: 3 to 7 points	27 (30 %)	22 (69 %)
Medium: 10 to 15 points	2 (2 %)	2 (6 %)
Large: >15 points	2 (2 %)	2 (6 %)
Total	90	32
Odds ratio		
0.95 – 1.05 or 1.0 – 1.05	35 (39 %)	0
0.90 – 0.95 or 1.05 – 1.10	33 (37 %)	7 (33 %)
0.80 – 0.90 or 1.10 – 1.20	19 (21 %)	11 (52 %)
<0.80 or > 1.20	3 (3 %)	3 (14 %)
Total	90	31

^a^MDs or ORs statistically significant at the 5 % level

Additional file 2: Figure S2 shows the relationship between MDs and ORs and shows general agreement in terms of the direction of effects (i.e. observations in the upper right quadrant are ORs >1 and MDs >0; estimates in the lower left are ORs < 1 and MDs <0).

Four examples are provided to understand the relationship between ORs and MDs.

### Example 1: when MDs are small but ORs are large

In the TOPICAL Trial the MD for constipation (CO) symptoms were 2.6 points (*p* = 0.1314) while this was an OR of 1.17 (*p* < 0.0001) – the choice of interpretation is ‘a worsening in CO by a mean difference of 2.6 points with erlotinib compared to placebo’ vs ‘patients are 17 % more likely of having worsening CO symptoms with erlotinib compared to placebo’. The MD scale gives the impression that CO symptoms worsens by a ‘Trivial’ amount of 2.6 points (Table 2). This tends to occur when responses are skewed (Fig. 1 and Additional file 2: Figures S1, S2 and S3). In the presence of heavily skewed data, the OR is a suitable choice for presenting HRQoL effects from the QLQ-C30.

### Example 2: when MDs are ‘Large’ but ORs are ‘Medium’ or ‘Small’

In the TOPICAL trial, patients had worse diarrhoea (DI) with erlotinib: MD of 15.1 (‘Large’ effect) points (*p* <0.001) with a corresponding OR of 1.12 (*p* = 0.0505). The DI scores were considerably skewed (Fig. 1) which might explain why the larger MD corresponded with only 12 % (‘Medium’ effect) higher odds of diarrhoea with erlotinib compared to placebo (OR = 1.12). The OR appears to have modified the ‘Large’ effect size (borderline significance) to a smaller (non-significant) effect size.

### Example 3: when MDs are ‘Medium’ but ORs are ‘Large’

In study 10, RF improved by a MD of about 13 points (Table 2) with the experimental treatment – a ‘Medium’ effect. Using an OR, this was an improvement in role function by almost 30 % (OR =1.29 ‘). On examination of Additional file 2: Figure S1, responses fell into only three distinct categories at 0, 50 and 100 and scores were not Normally distributed making use of the MD questionable. The OR approach has relegated a ‘Medium’ effect to a ‘Large’ effect.

### Example 4: when MDs and ‘ORs agree on the direction of effects

In the TOPICAL trial, two of the MDs (MD of 3.2 and 3.6 in TOPICAL; *p*-values of 0.0017 and 0.0007 for PF and CF respectively) had corresponding ORs of 1.10 and 1.14 (*p*-value = 0.0168 and 0.0107). Both MDs and ORs are in agreement that PF and CF are improving with the experimental treatment. Hence, on average, patients had 10 % and 14 % higher odds of improved PF and CF on erlotinib compared with placebo respectively (Table 2).

The above are a limited number of examples reflecting the challenges associated with defining thresholds of HRQoL differences with the MD. Another issue that can complicate interpretation is when small effects become difficult to interpret and justification is made through statistical significance. Statistical significance of small HRQoL effects are often reported, but the clinical relevance not always discussed. Table 3 shows that 28/90 (31 %) of ‘small’ or ‘Trivial’ effects based on MD were statistically significant compared with 7/90 (8 %) for ORs.

### Example 5: Potentially unreliable statistically significant conclusions using MD

In study 12, for Diarrhoea, the MD was −2.3 (*p* = 0.0017). The corresponding OR was 1.05 (*p* = 0.2909). The clinical relevance of the small improvement in DI symptoms with experimental treatment might be difficult to judge. On the ORs scale, DI is actually shown to be worse: a 5 % likelihood of worsening diarrhoea (a common side effect with this chemotherapy) on the experimental treatment. Examination of Additional file 2: Figure S2 shows heavily skewed DI scores – with about 15 % of patients showing worsening DI symptoms. The choice of a mean statistic here is likely to lead to an unreliable or unexpected statistical conclusion. Further examples of differing statistical conclusions between ORs and MDs are shown in Additional file 2: Tables S2, S3.

### Effect size classification for ORs and MDs

**Fig. 2:**
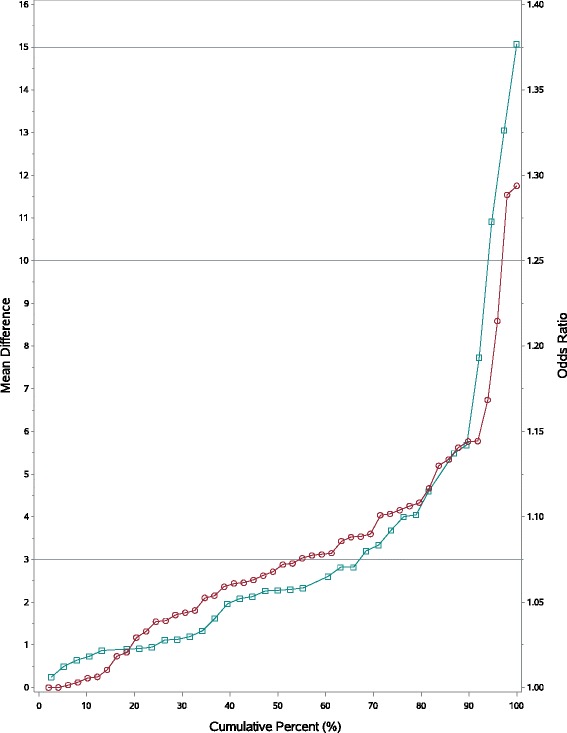
Cumulative Frequency Plot of Effect Sizes for MDs and ORs. Horizontal reference lines are MDs effect sizes of 3, 10 and 15 points; circles refer to ORs and squares refer to MDs

Estimates for OR effect size categories similar to those described earlier [7] were determined using a cumulative frequency plots from MDs and ORs (Fig. 2 and Additional file 2: Tables S2, S3, S4). Effect sizes in terms of ORs were broadly classified as: ‘Trivial’: ORs within ±5 % of 1 (i.e. ORs between 0.95 and 1.05); ‘Small’ effects (ORs 1.05 -1.10 or 0.90 – 0.95); ‘Medium effects (ORs 1.10 – 1.20 or 0.80-0.90) and ‘Large’ effects ORs either >1.20 or <0.80. Additional file 2: Table S4 shows that 12/59 (20 %) of ‘Trivial’ effects based on MDs might be clinically important because on an OR scale these were ‘Medium’ or ‘Large’. Consequently some clinically important effects may be missed using MDs.

Figure 2 shows median HRQoL effect sizes are 2.5 points (half of effect sizes are ≤2.5), roughly equivalent to 7 % changes in HRQoL on the OR scale; similarly for the lower and upper quartiles, 25 % of effect sizes ≤1 point or 4 % changes on the OR scale; and 75 % of effect sizes are ≤3.6 points (ORs of about 1.10).

Secondly, for effect sizes of 1, 3, 5 10 and >15 points, the equivalent ORs are about 1.02, 1.07, 1.13, 1.25 and 1.37 respectively. The threshold for a large effect size of >15 points is challenging: patients expected to improve/worsen by almost 40 %. This may be a difficult target for some cancer drugs to achieve when compared with each other.

### Summary of preference scores from survey

Five lung cancer clinicians completed a pilot (Additional file 1) survey (London UCH, Liverpool, Leeds, Chester and Imperial College London). At this time no patient responses were available. Hence a total of 15 scores from 5 clinicians who expressed preferences for either ORs or MDs for each of PF, Pain and CF were analysed. Stronger preferences were expressed for ORs over MDs: mean scores of 2.4, 3.1 and 2.8 for PF, Pain and CF respectively. Hence, initial evidence suggests clinician preference was greater for ORs than MDs. The results would need to be confirmed in a larger sample.

### Comparison with time to deterioration

The time it takes for a patient to deteriorate from baseline by ≥5 was not possible for about 13 % HRQoL domain scores due to too few events (i.e. patients did not show of ≥5 points). Moreover, a TD of ≥5 points was not always possible because scores were clustered in values such as 16.7, 33.3 and 66.6 (e.g. as in CF scores for TOPICAL -Fig. 1). No patient experienced (or could experience) a TD of exactly 5, 10 or 15 points (the possible values of the QLQ-C30 for CF were only 0, 16.7, 33.3, 50.0, 66.7, 83.3 and 100). The median TD (Additional file 2: Table S5) was not calculable for some symptom and function scores: for CF, a HR of 1.05 (*p* = 0.241) was reported: patients had a 5 % increased risk of deteriorating (≥5 point reduction) CF with erlotinib compared to placebo. The OR of 1.14 and MD of 3.2 in contrast show improvements in CF. The definition of deterioration is therefore critical for a valid estimate to be possible. When the TD for CF was changed to ≥16 points (‘Large’ effect), the medians become calculable as 77 vs 87 months for erlotinib vs placebo (HR = 0.92; *p* = 0.56): the risk of deterioration in CF was slightly worse (by 8 %) with erlotinib compared to placebo. The Kaplan Meier curves cross and the PH assumption was violated, a complication the OR analysis avoids.

## Conclusion

An alternative metric to the commonly reported MD was presented in the form of ORs. Skewness of QLQ-C30 scores might render statistical and clinical interpretation of MDs questionable. Alternative effect size categories for ORs were proposed. We have also shown a relationship between ORs and MDs for QLQ-C30 measures; ORs can on the one hand reveal important HRQoL effects which might otherwise be missed with MDs, particularly those perceived to be ‘Trivial’ or ‘Small’. Conversely, effect sizes based on MDs thought to be ‘Medium’ or ‘Large’ may appear less exaggerated with ORs; Treatment effects from TD type analyses did not always result in estimates of effect sizes and interpretations were complicated by non PH assumptions. Finally we showed results from a pilot survey which suggest oncologists may prefer ORs over MDs for interpreting QLQ-C30 effects.

The use of the ORs has been used previously in HRQoL data. Feddern et al. (2015) [27] reports them for assessment of pain; Chie et al. (2015) [28] uses a propensity score (logistic regression) approach to report odds of HRQoL deterioration; Kurita et al. (2015) [29] use ORs with the QLQ-C30 in renally impaired patients. In these analyses scores were dichotomized in order to generate the ORs. In our analysis, no such dichotomization (and consequent loss of information) was required due to flexibility of the Beta-Binomial regression approach.

Patient and clinician understanding of MDs have not been previously shown to be concordant [7] and this may in part be due to how HRQoL benefits are expressed to patients. Clinicians and patients may find it easier to agree on relative quantities than absolute differences. The pilot survey results may support relative quantities. The choice between interpretations such as: “your diarrhoea will be worse with the new treatment by 15 points, on average” instead of: “the likelihood of diarrhoea with the new treatment is significantly higher by about 11 % compared to placebo”, is a matter of preference, but the latter may be appealing for some. Aligning understanding of smaller effect sizes is increasingly important with the emergence of novel treatments for lung cancer being compared with each other (and not just placebo).

There are several advantages and disadvantages of both MDs and ORs. First, ORs evaluate relative (instead of absolute) treatment effects. For objective endpoints, absolute differences (e.g. 4 vs 3 months survival) may provide easier interpretations of treatment benefits (although the effects are median and not mean differences in cancer trials). However, HRQoL are self-reported endpoints for which even the most experienced clinician has difficulty interpreting. For such endpoints, a relative scale may be more useful. If treatment effects from primary endpoints are judged by relative quantities (e.g. hazard ratios), there are no reasons why treatment effects from HRQoL endpoints should not also be assessed this way. Both survival time and HRQoL share some similar distributional properties (e.g. skewed or censored). There is some concern that effects near the boundaries (floor/ceiling) will be overvalued with ORs compared to effects around the middle. However, such concerns can be addressed through the use of zero–one inflated models (Khan, 2014) [25] which model the over/under dispersion.

Secondly, the OR model assumes a fixed odds ratio over time (i.e. the effect is constant over time), which may not hold in a longitudinal QoL setting. Reliable interpretation of MDs also depends on an absence of treatment by time interactions (i.e. ORs and MDs are not dependent on specific time points). Thirdly, statistical models for MDs will provide predicted patient level HRQoL responses. For example, a patient taking experimental treatment with a certain demographic profile might yield a predicted PF score (e.g. 5 points). Similarly, a model for estimating ORs can be used to predicted a probability of a achieving a specific PF score for a given patient (group of patients) on the experimental treatment (response curves are advocated by the FDA for patient reported outcomes) [30].

The suggested effect size of >10 units on the QLQ-C30 was proposed almost two decades ago when fewer treatment comparators were available [15]. Few (about 2 %) MDs were >10 points and this research confirms earlier conclusions that small changes in HRQoL can be important (Cella, 2002) [7, 31]. Importantly, the implications of skewed distributions were not factored in when the magnitude of effect sizes were defined in earlier research.

There are several strengths and limitations of this analysis. First, a large sample size is used from clinical trials in similar groups of patients. Secondly, established criteria for classifying effect sizes were used for MDs [7]. Third, the BB model is a robust approach to analysing skewed data with ceiling effects, without arbitrary dichotomisation of responses. Finally, interpreting ORs is similar to that of HRs which many oncologists are familiar with.

Although the BB approach offers an alternative approach to analyse and interpret HRQoL effects, it is more complex. The complexity is outweighed by the benefits of reliable and potentially easier to interpret estimates of effect. A further limitation is that analysis has been restricted to lung cancer patients, but can be applied to other tumour types and disease areas. The classifications suggested for ORs in this analysis are arbitrary (even if based on the observed data) and different results can occur with alternative categories. Definition of effect sizes may require some threshold to be set which may necessarily be subjective. However, a starting point in our view is that the most appropriate metric is used to present HRQoL effects in cancer patients, an area for further research. The initial survey results too should also be confirmed in a larger sample size.

Treatment effects for HRQoL from the QLQ-C30 should be reported using relative quantities such as ORs which appear to be clinically intuitive, easier to interpret and where analysis involves modelling the skewed distribution of responses.

### Highlights

The highlights of this paper are:

- Mean differences in HRQoL are difficult to interpret for clinicians and patients alike, especially when the difference is small.
- An alternative measure to reporting and interpreting HRQoL treatment differences using a relative quantity such as an odds ratio can greatly facilitate patient –clinician understanding of a ‘relevant’ HRQoL improvement.
- We offer a way in which mean differences in HRQoL can be interpreted as approximate odds ratios. Effect sizes are categorized as ‘Trivial, ‘Small’ ‘Medium’ and ‘Large’ for odds ratios in a similar way to mean differences
- Although the BB approach offers an alternative approach to analyse and interpret HRQoL effects, it is more complex. The complexity is outweighed by the benefits of reliable and potentially easier to interpret estimates of effect.
- Our approach will allow patients and clinicians to align their understanding of treatment benefits using HRQoL outcomes.

## Additional files

Additional file 1:
**Short survey questionnaire.** (DOC 45 kb) Additional file 2:
**Supplementary tables and figures.** Figure S1: Distribution of QLQ-C30 responses. a)SOCCAR, b)Study 10, c)Study 11, d)Study 12, e)Study 14. (x-axis is QLQ-C30 score on a scale of 0 to 1 and - y axis is relative frequency). Figure S2: Plot of Odds Ratios vs. MDs for all 15 domains (all trials). a) Overall, b) Functional Domain, c) Symptom Domain. Vertical reference lines are ±10 points for MDs and zero (no effect line). Horizontal reference lines are 1 (no effect) and 0.8 and 1.20 for ORs. Figure S3(a): Comparison of erlotinib vs placebo responses for EF in TOPICAL trial. (b): Comparison of erlotinib vs placebo responses for CO in TOPICAL trial. Example showing MDs not statistically different but ORs statistically significant: Higher proportion of placebo responses for lower scores and higher proportion of erlotinib responses in some categories of emotional function (EF) >0.6 (or 60); distribution is skewed. (DOC 955 kb)
