# Supplementary material for: Interpreting small treatment differences from quality of life data in cancer trials: an alternative measure of treatment benefit and effect size for the EORTC-QLQ-C30
Source: Health Qual Life Outcomes. 2015 Nov 14;13:180. doi: 10.1186/s12955-015-0374-6 (PMC4647515; doi:10.1186/s12955-015-0374-6)
Supplement: Additional file 1: — Short survey questionnaire. (DOC 45 kb) [file 12955_2015_374_MOESM1_ESM.doc]

**Supplementary Appendix I: Short Survey Questionnaire**

Patient/Clinician Number: ________________

Patient Clinician (tick appropriate responder)

_________________________________________________________

Each of the *two* statements below are describe ways of measuring *how much* a new treatment might make a patient feel better or worse compared to their current treatment.

You are asked to show which of the two statements are preferred by marking an “X” on the line below.

(1)

1. “Your Physical Function is likely to improve by 20% on average with the new treatment compared to the current treatment”
2. “Your Physical Function will improve by 10 points on average with the new treatment compared to the current treatment”

Prefer (a) No Preference Prefer (b)

(2)

1. “Your Pain is likely to worsen by 15% on average with the new treatment compared to the current treatment”
2. “Your Pain will get worse by 6 points on average with the new treatment compared to the current treatment”

Prefer (a) No Preference Prefer (b)

(3)

1. “Your Cognitive Function is likely to improve by 5% on average with the new treatment compared to the current treatment”
2. “Your Cognitive Function will improve by 2 points on average with the new treatment compared to the current treatment”

Prefer (a) No Preference Prefer (b)
