# Supplementary material for: Interpreting small treatment differences from quality of life data in cancer trials: an alternative measure of treatment benefit and effect size for the EORTC-QLQ-C30
Source: Health Qual Life Outcomes. 2015 Nov 14;13:180. doi: 10.1186/s12955-015-0374-6 (PMC4647515; doi:10.1186/s12955-015-0374-6)
Supplement: Additional file 2: — Supplementary tables and figures. Figure S1: Distribution of QLQ-C30 responses. a)SOCCAR, b)Study 10, c)Study 11, d)Study 12, e)Study 14. (x-axis is QLQ-C30 score on a scale of 0 to 1 and - y axis is relative frequency). Figure S2: Plot of Odds Ratios vs. MDs for all 15 domains (all trials). a) Overall, b) Functional Domain, c) Symptom Domain. Vertical reference lines are ±10 points for MDs and zero (no effect line). Horizontal reference lines are 1 (no effect) and 0.8 and 1.20 for ORs. Figure S3(a): Comparison of erlotinib vs placebo responses for EF in TOPICAL trial. (b): Comparison of erlotinib vs placebo responses for CO in TOPICAL trial. Example showing MDs not statistically different but ORs statistically significant: Higher proportion of placebo responses for lower scores and higher proportion of erlotinib responses in some categories of emotional function (EF) >0.6 (or 60); distribution is skewed. (DOC 955 kb) [file 12955_2015_374_MOESM2_ESM.doc]

**Supplementary** **Figure 1: Distribution of QLQ-C30 responses**

1. SOCCAR

| 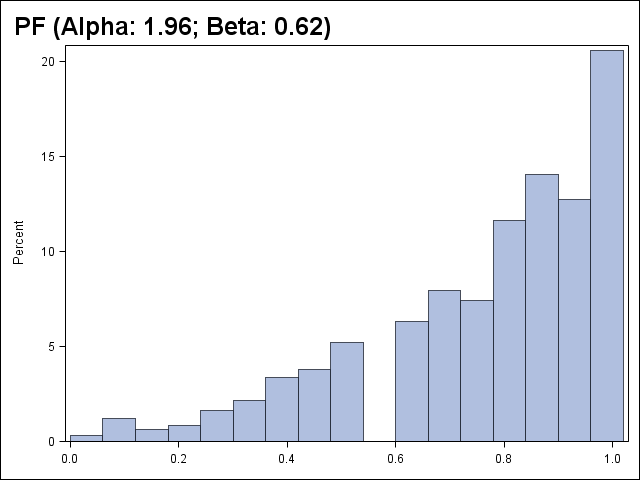 | 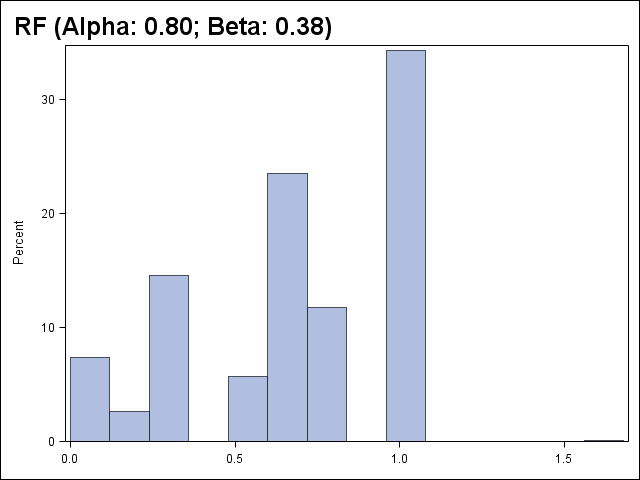 | 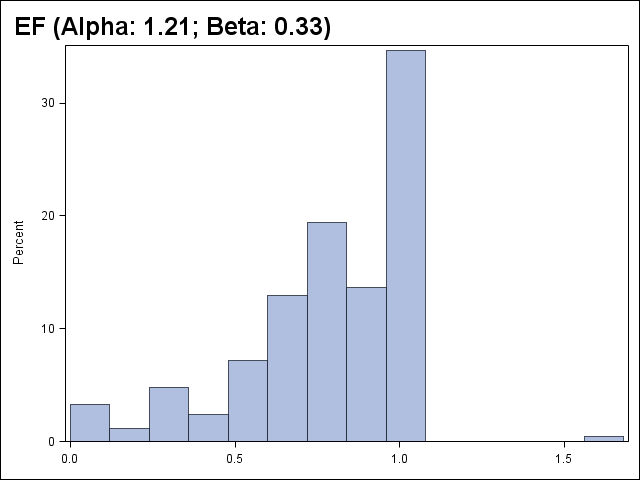 |
| --- | --- | --- |
| 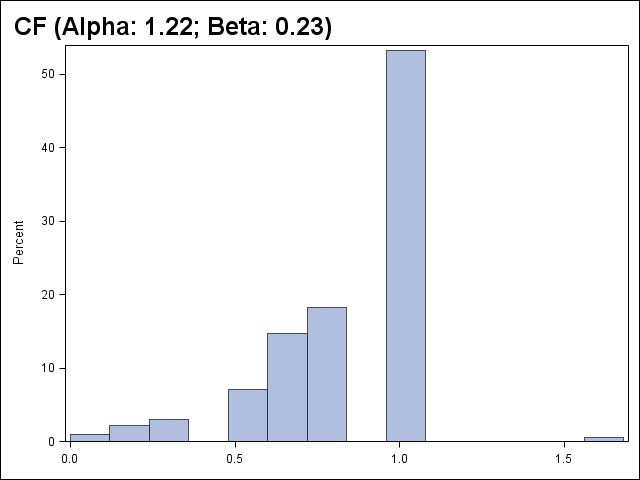 | 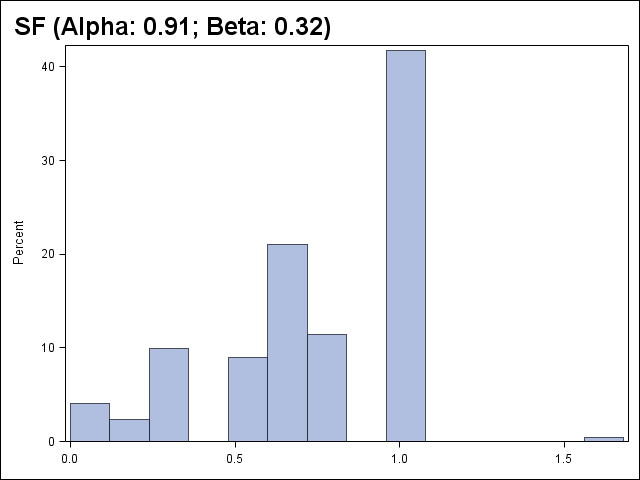 | 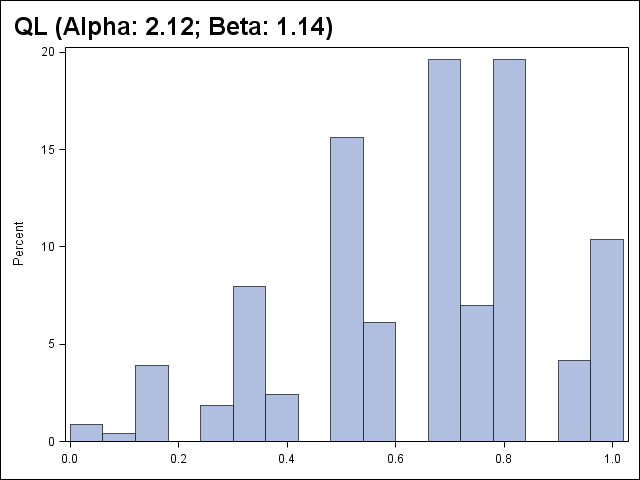 |
| 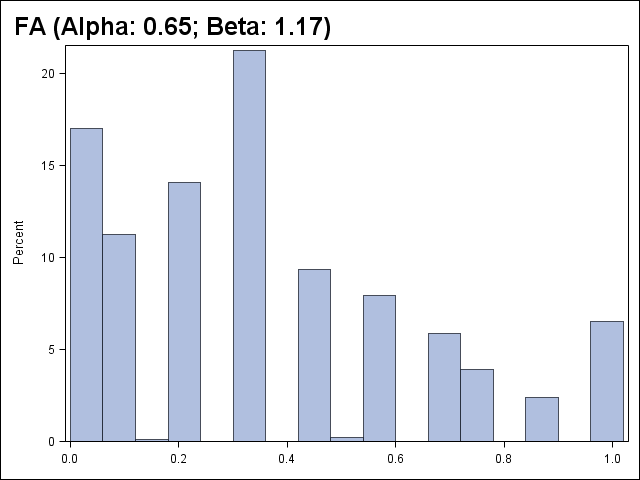 | 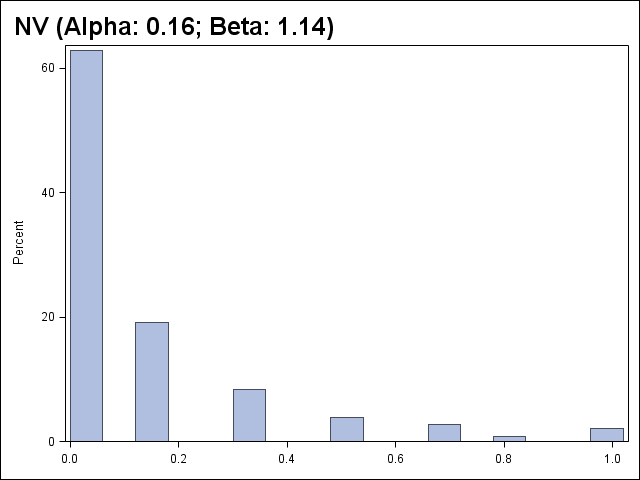 | 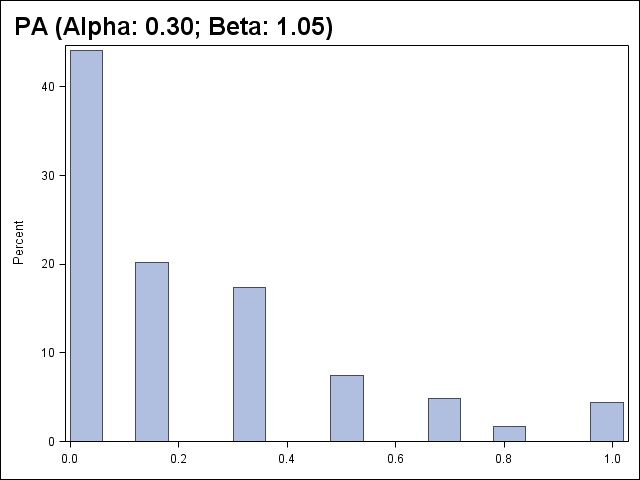 |
| 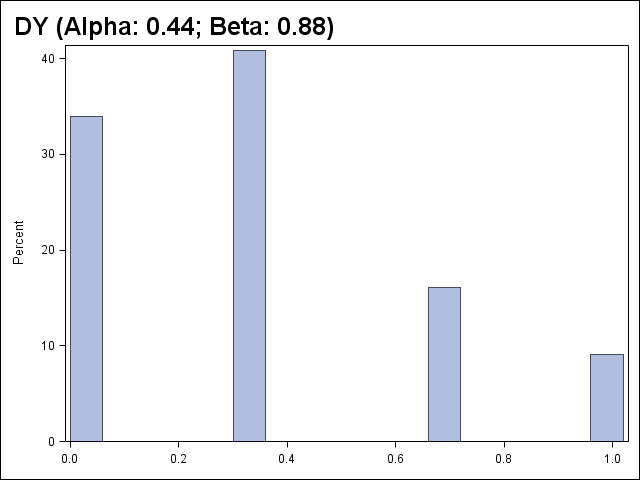 | 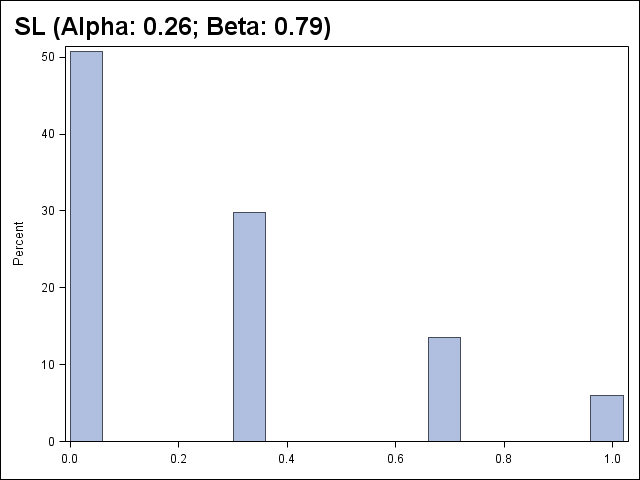 | 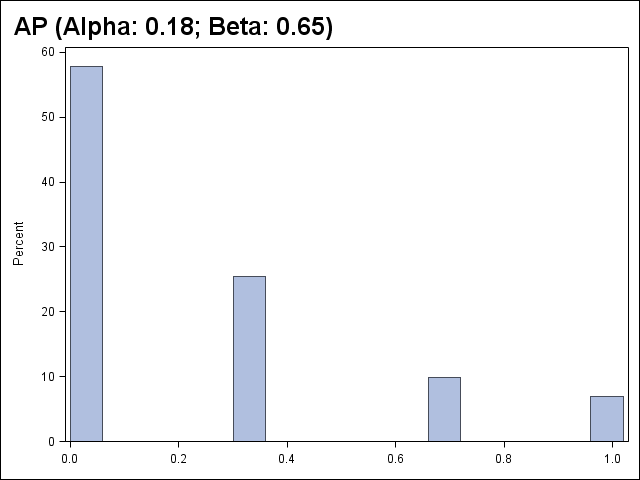 |
| 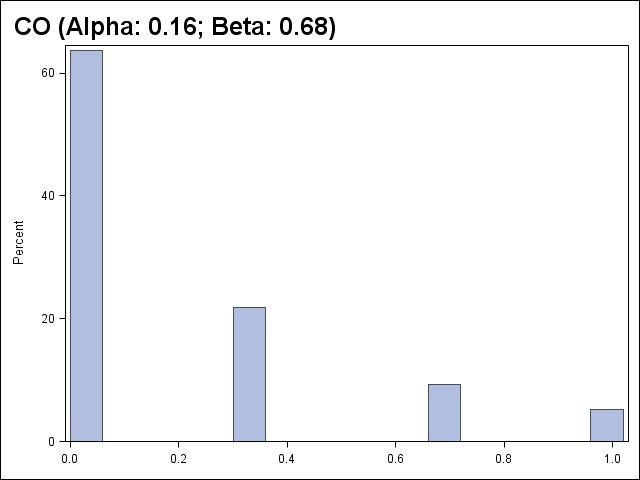 | 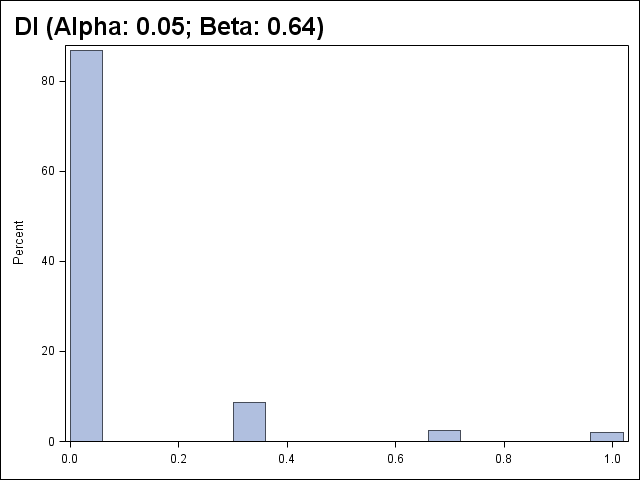 | 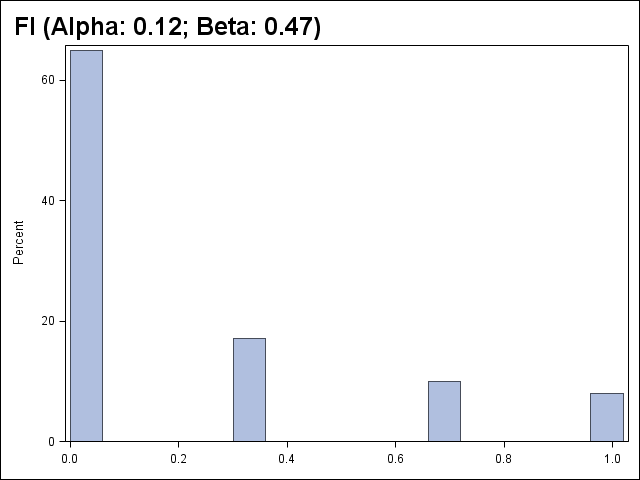 |

**(x-axis is QLQ-C30 score on a scale of 0 to 1 and - y axis is relative frequency)**

1. Study 10

| 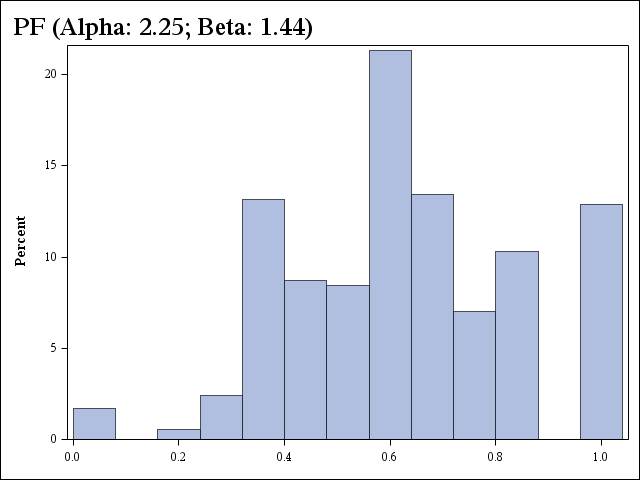 | 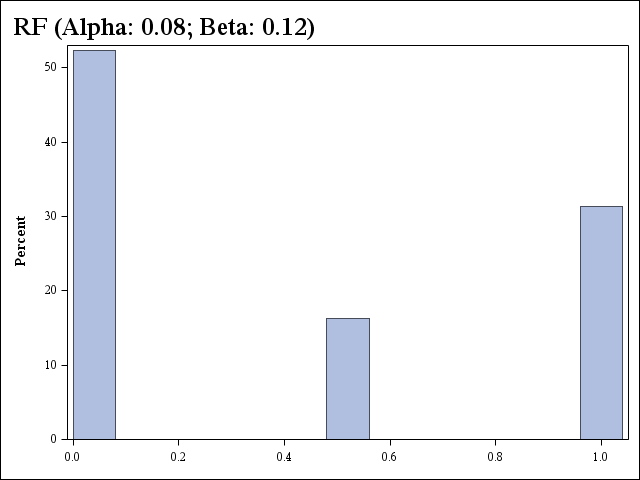 | 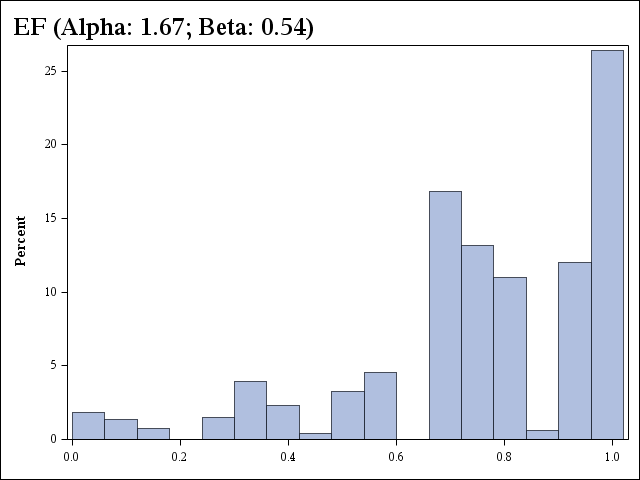 |
| --- | --- | --- |
| 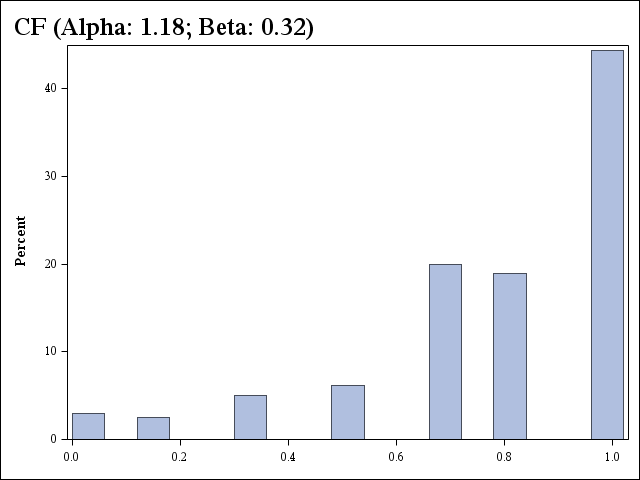 | 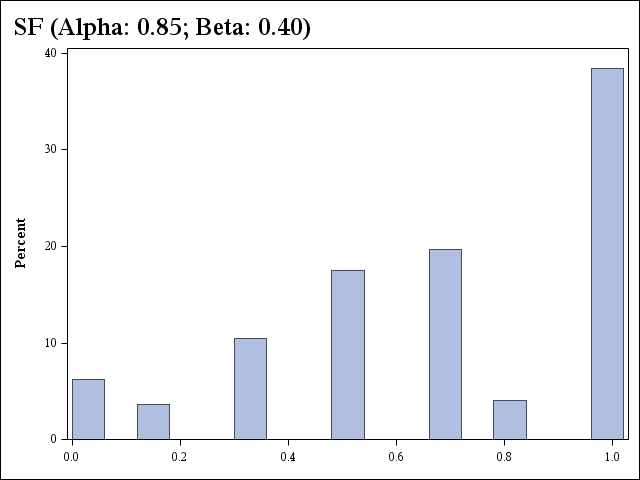 | 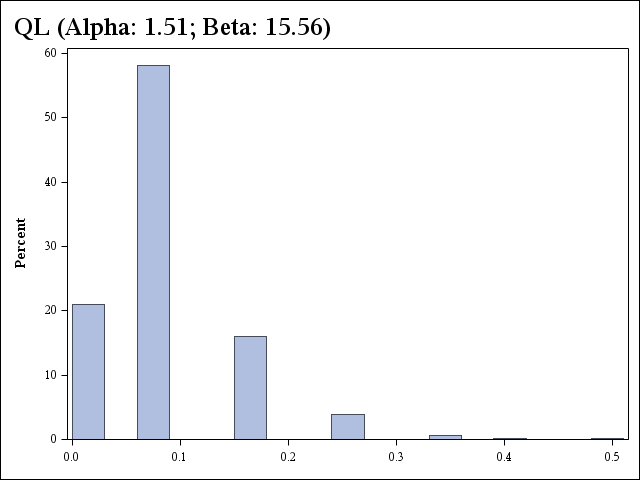 |
| 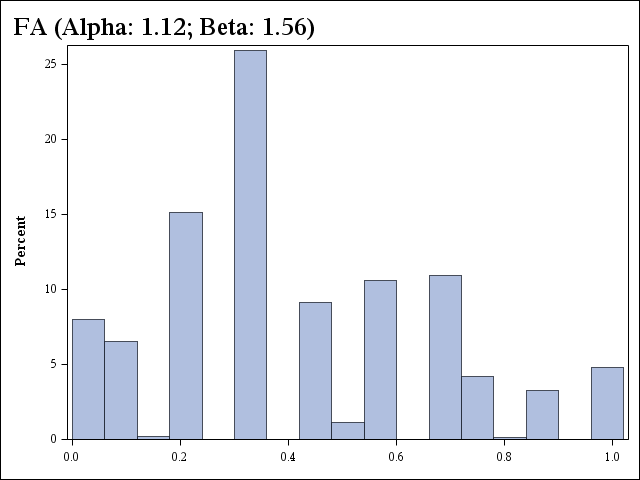 | 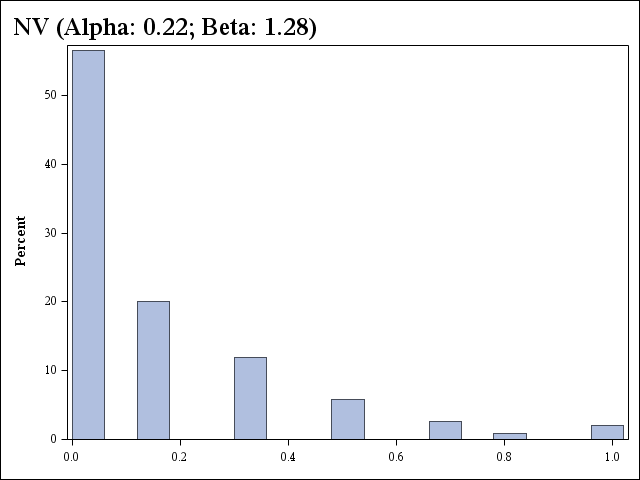 | 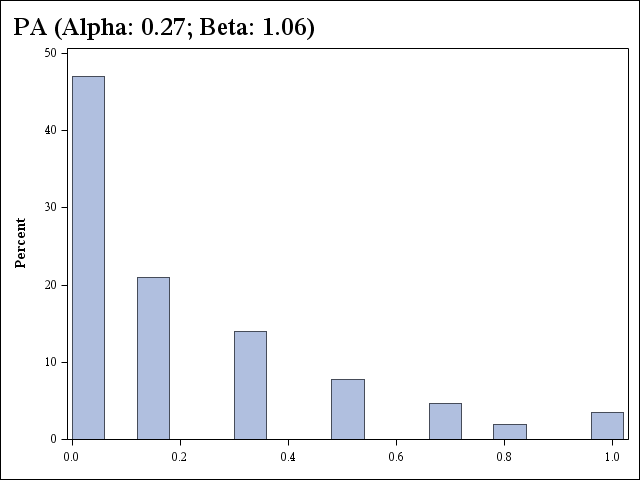 |
| 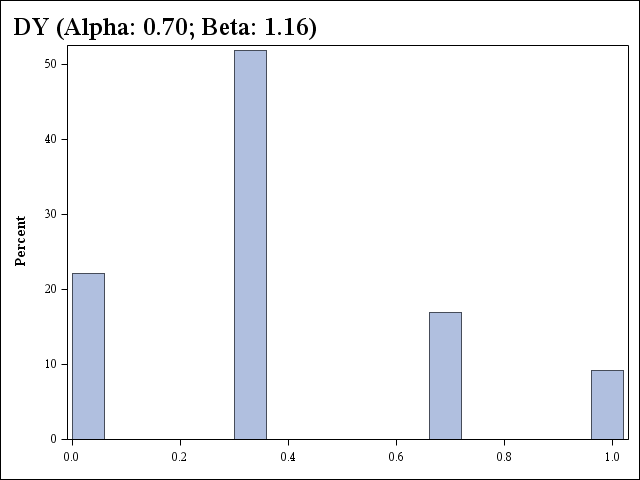 | 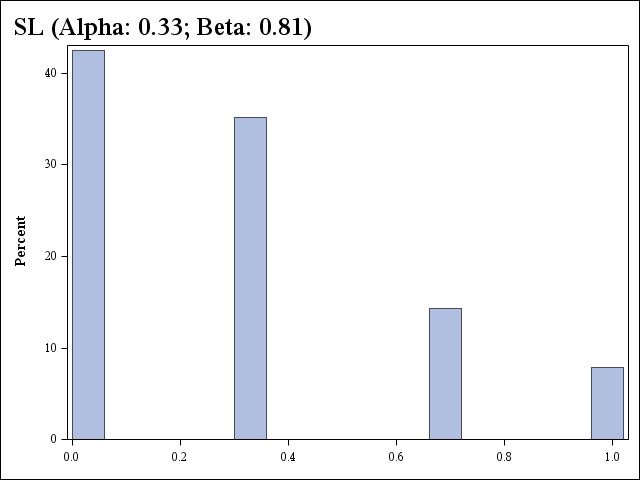 | 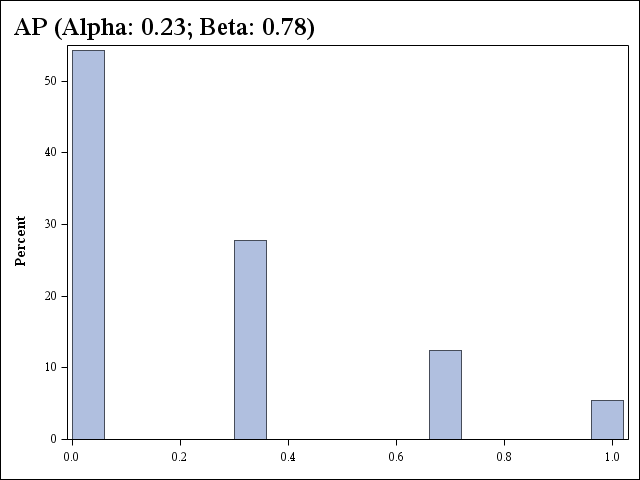 |
| 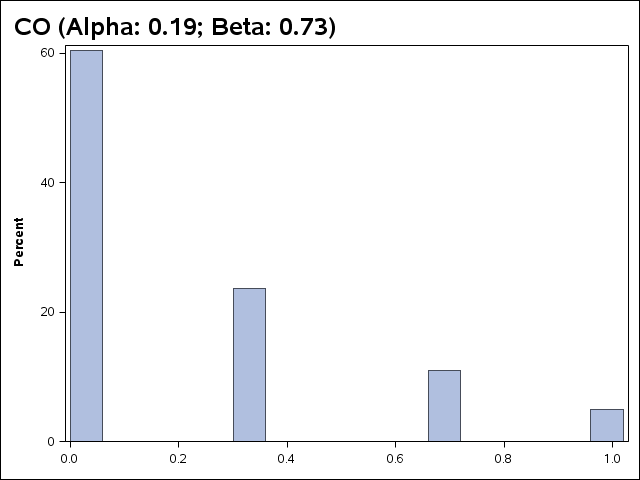 | 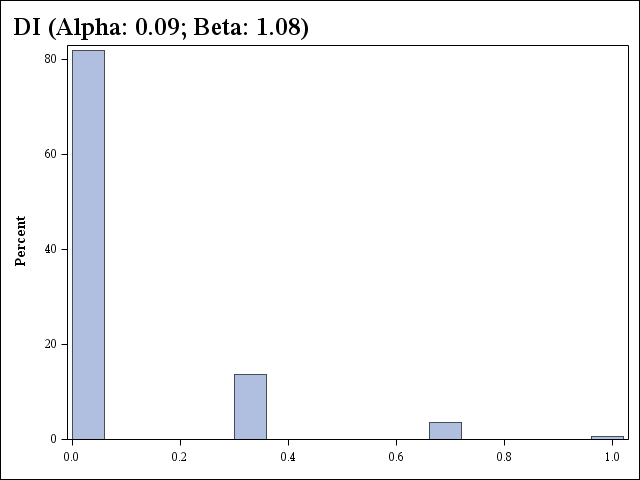 | 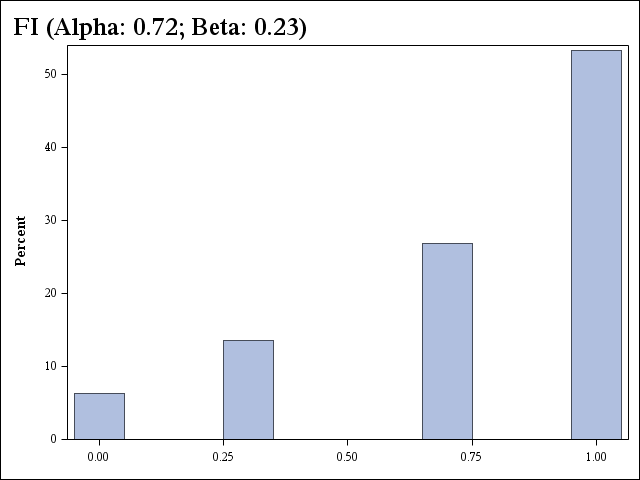 |

1. Study 11

| 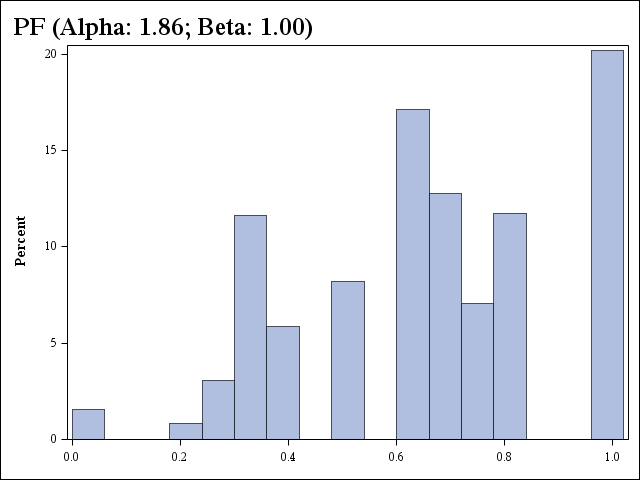 | 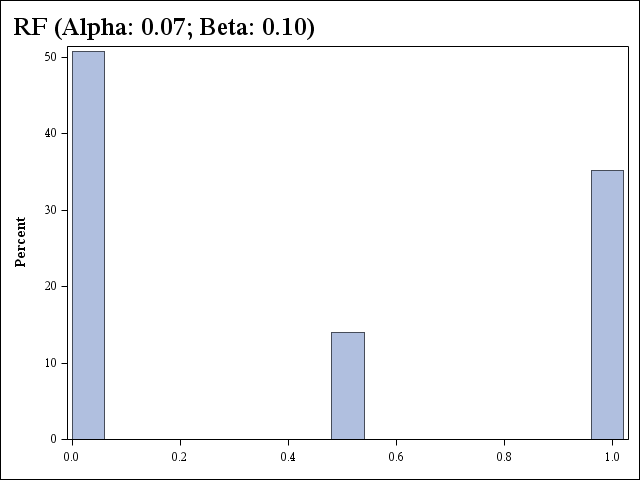 | 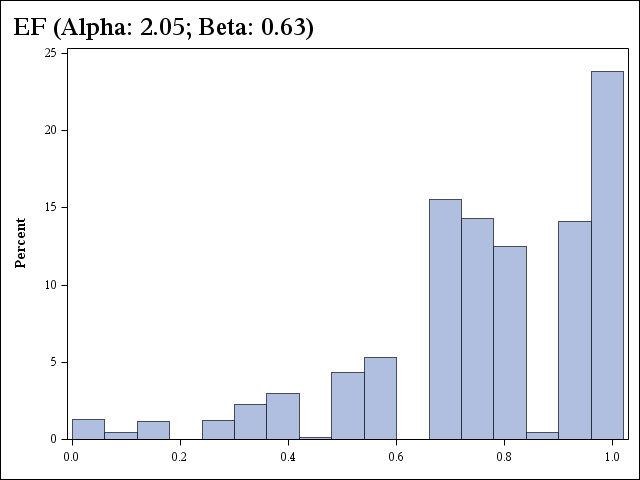 |
| --- | --- | --- |
| 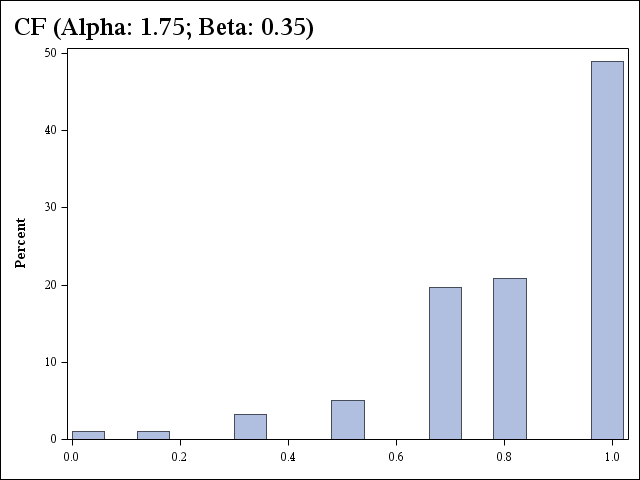 | 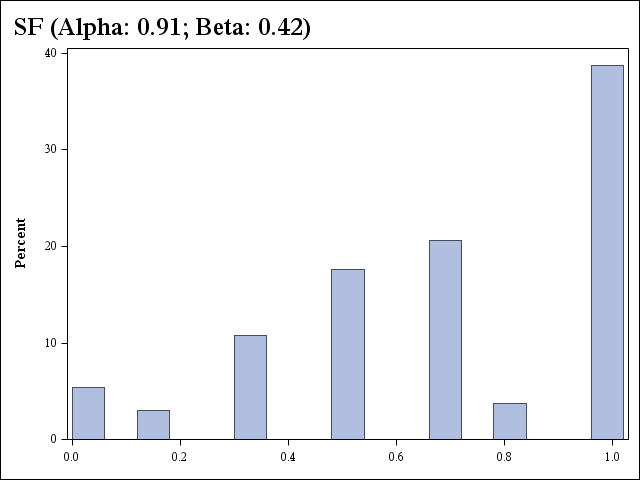 | 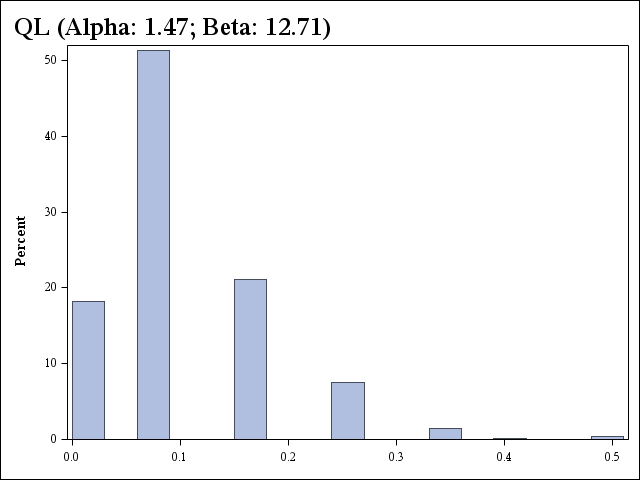 |
| 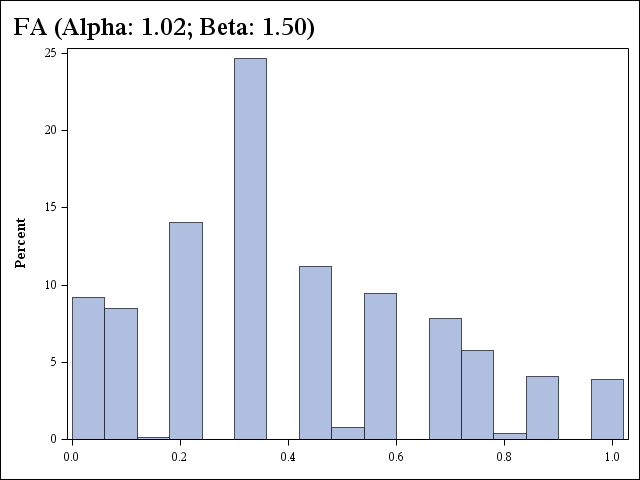 | 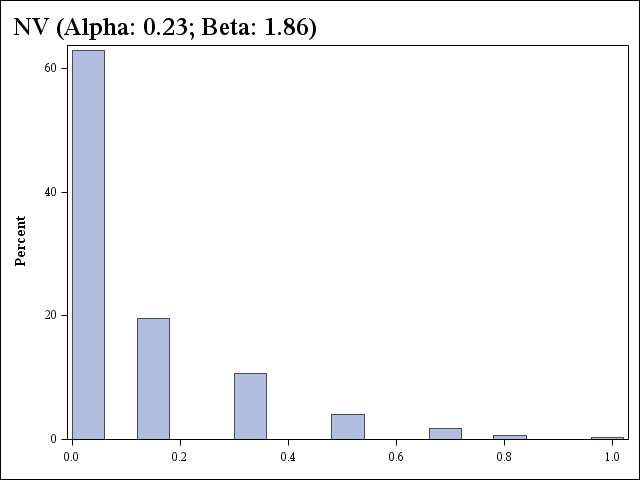 | 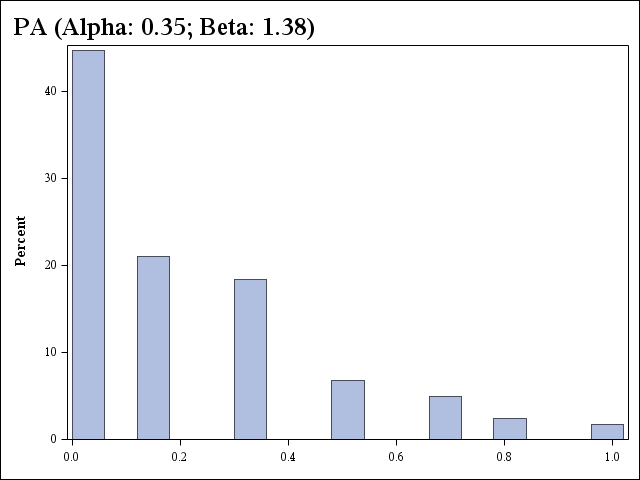 |
| 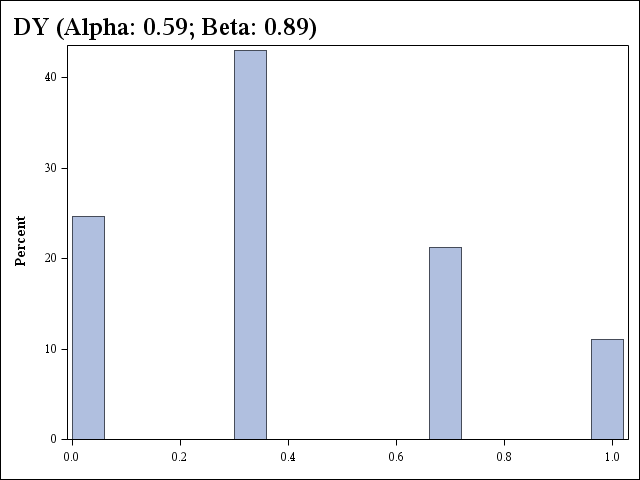 | 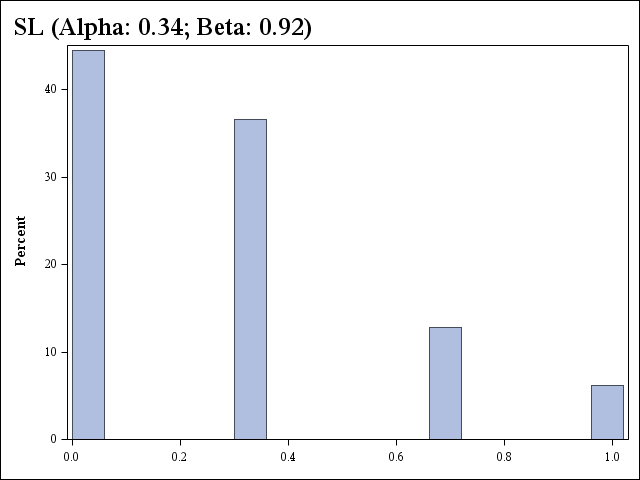 | 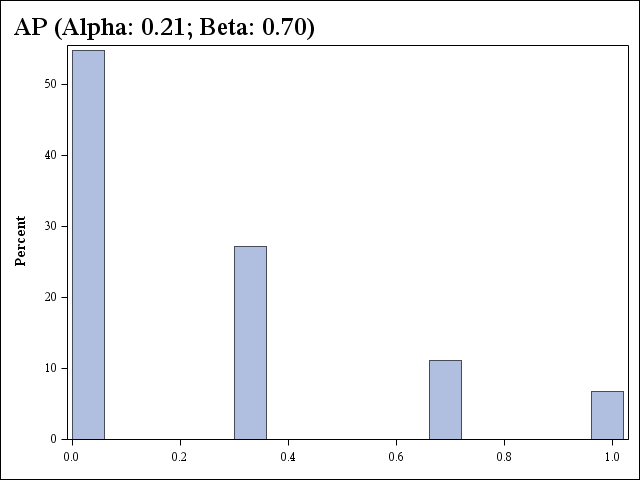 |
| 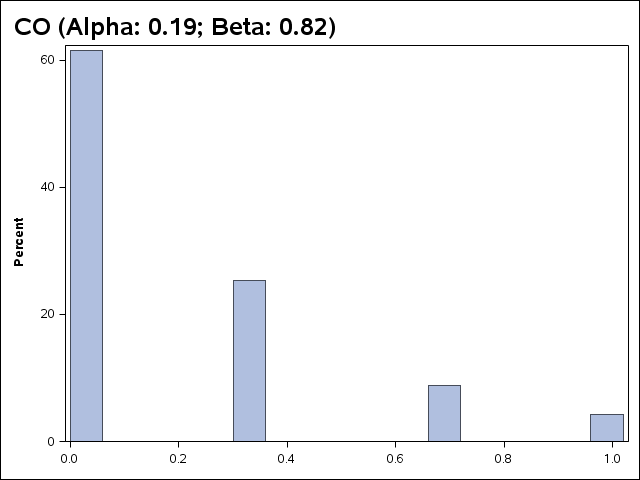 | 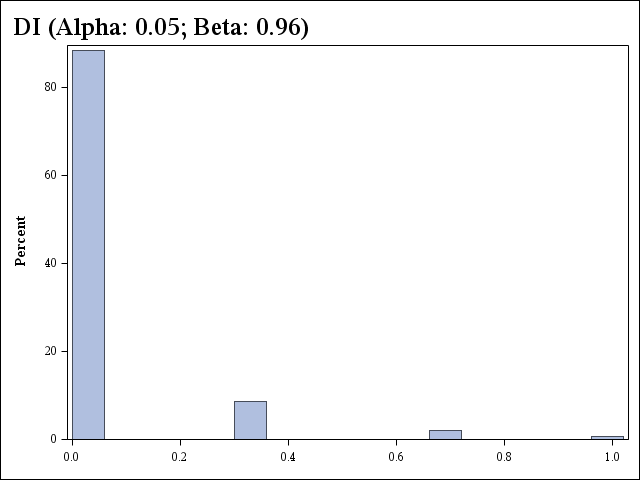 | 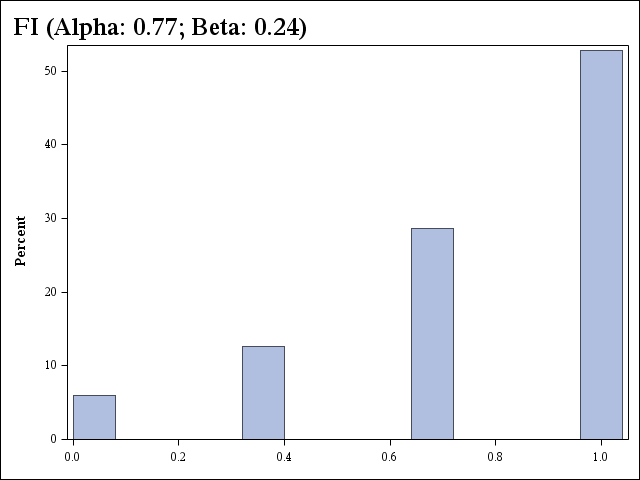 |

1. Study 12

| 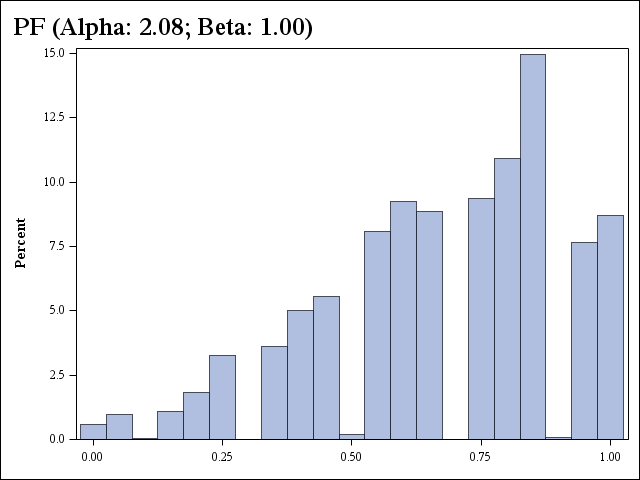 | 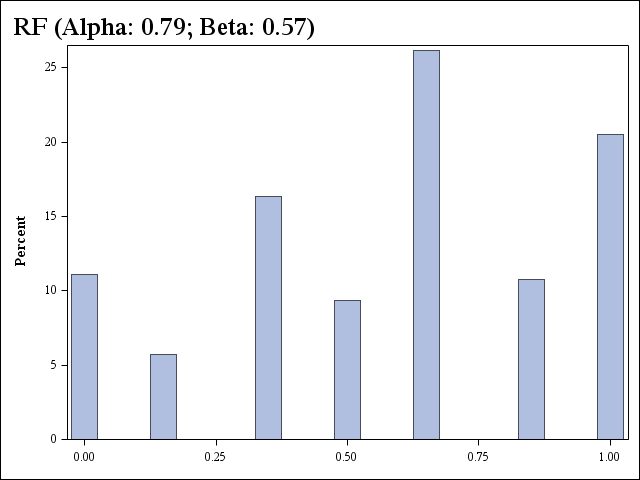 | 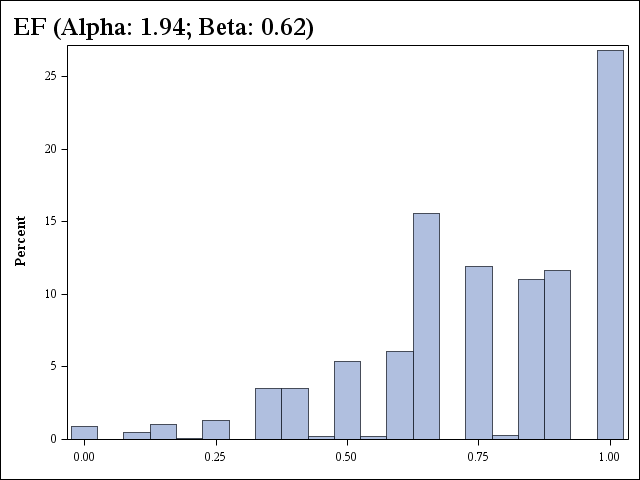 |
| --- | --- | --- |
| 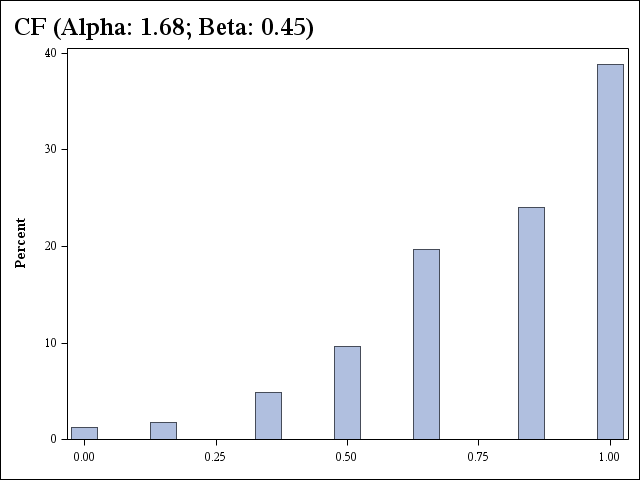 | 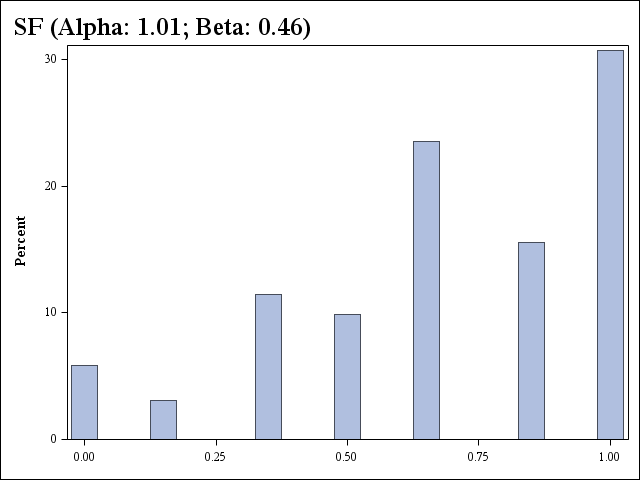 | 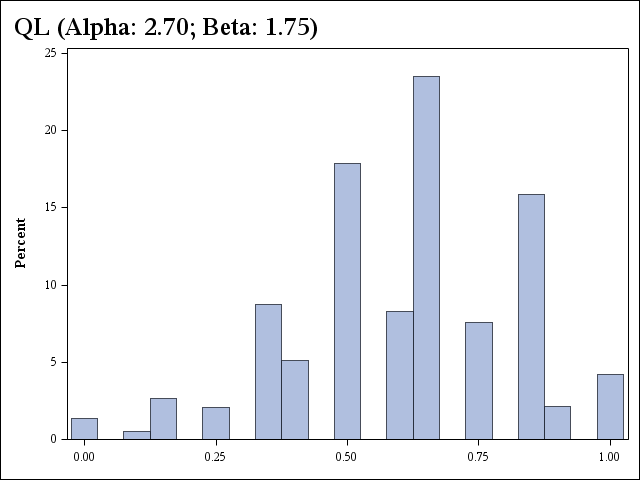 |
| 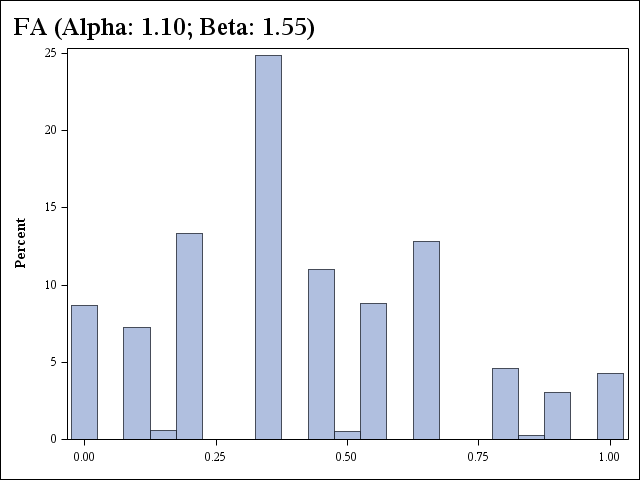 | 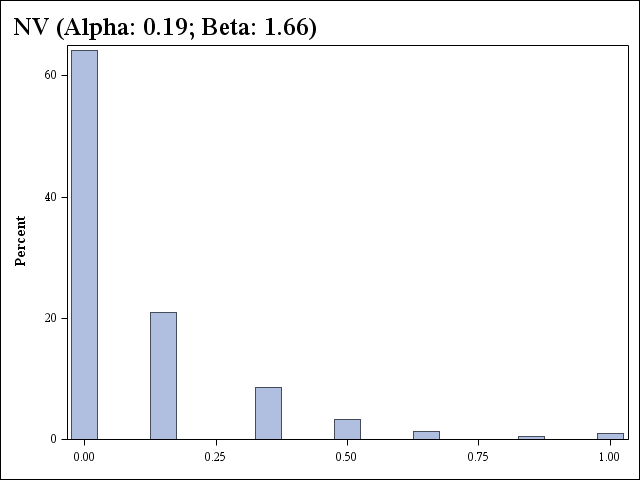 | 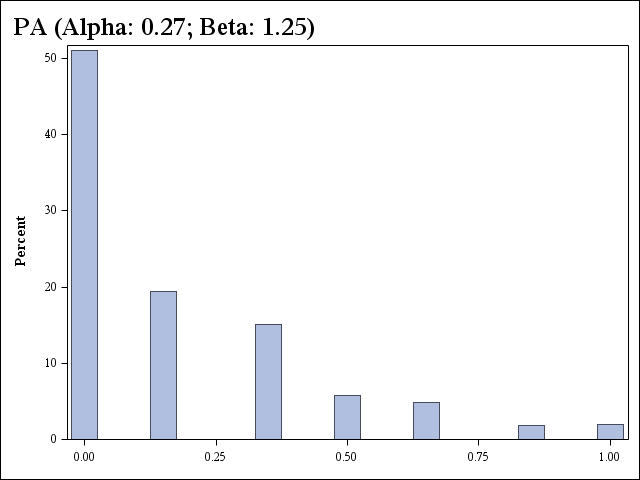 |
| 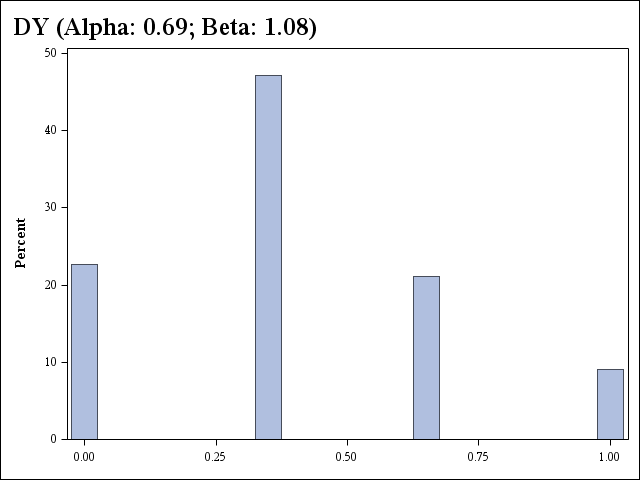 | 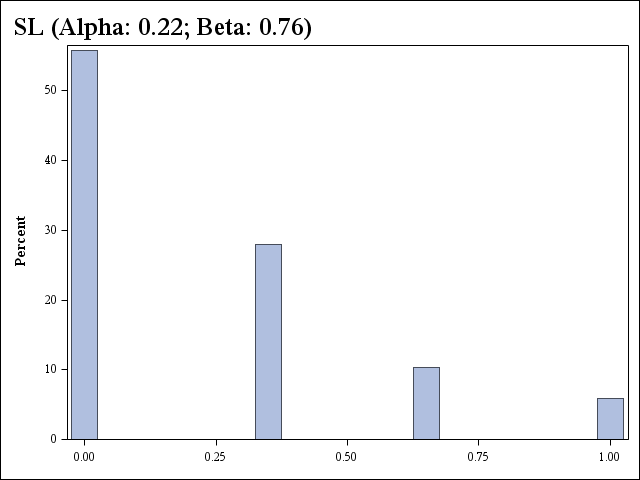 | 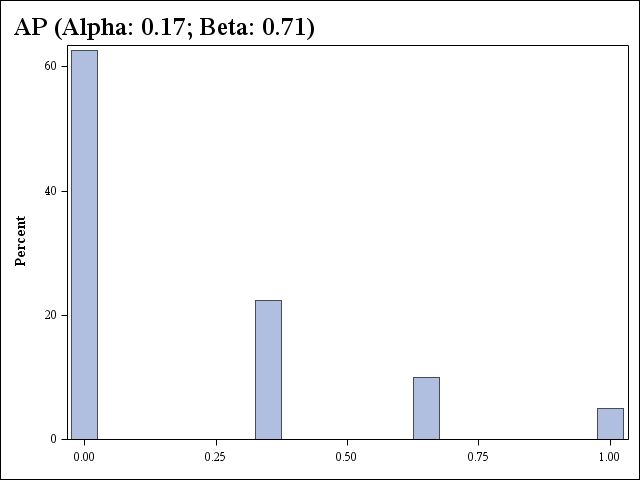 |
| 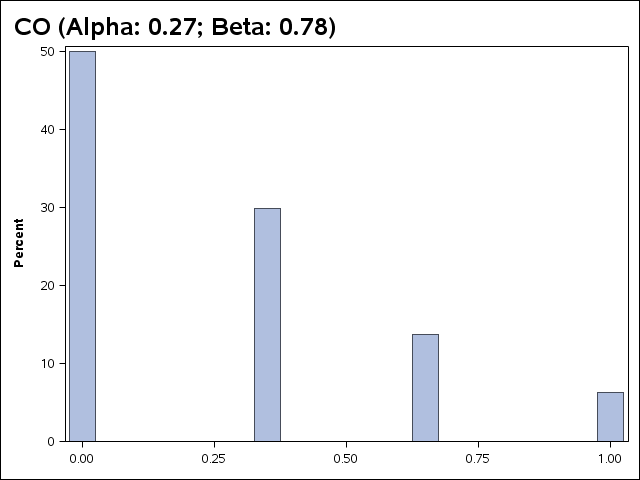 | 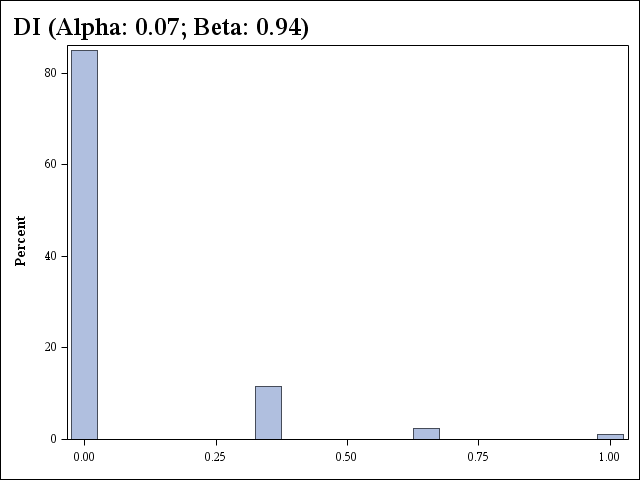 | 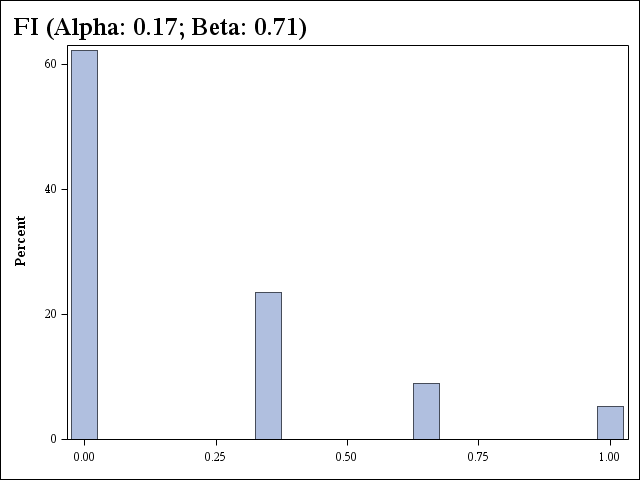 |

1. Study 14

| 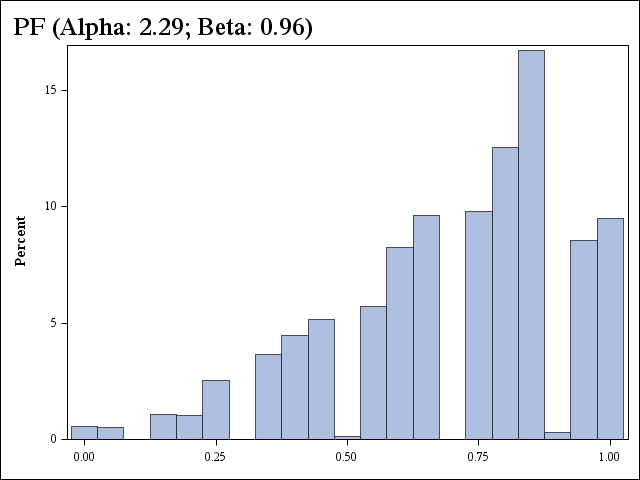 | 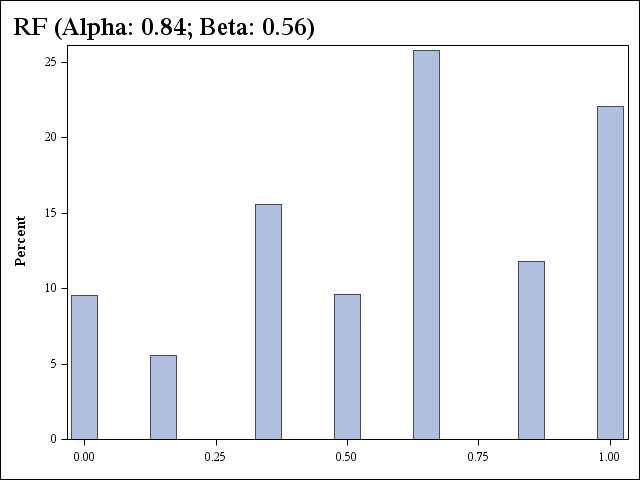 | 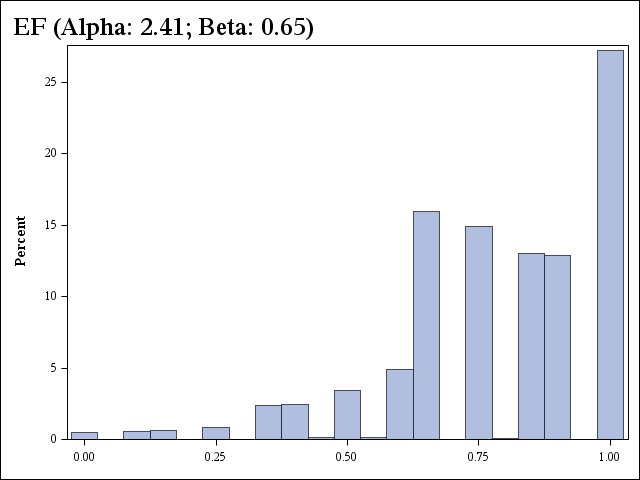 |
| --- | --- | --- |
| 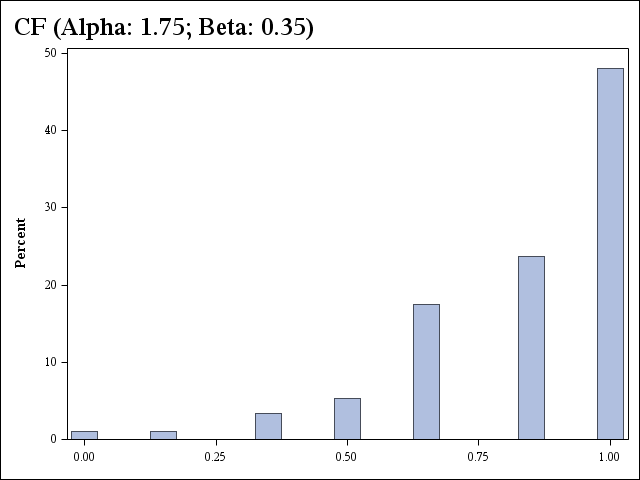 | 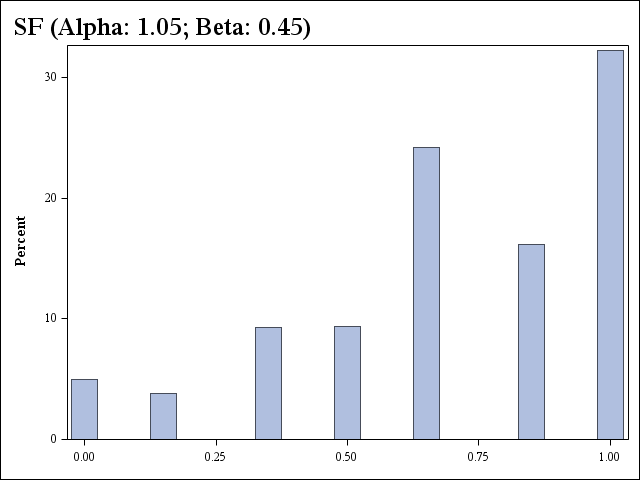 | 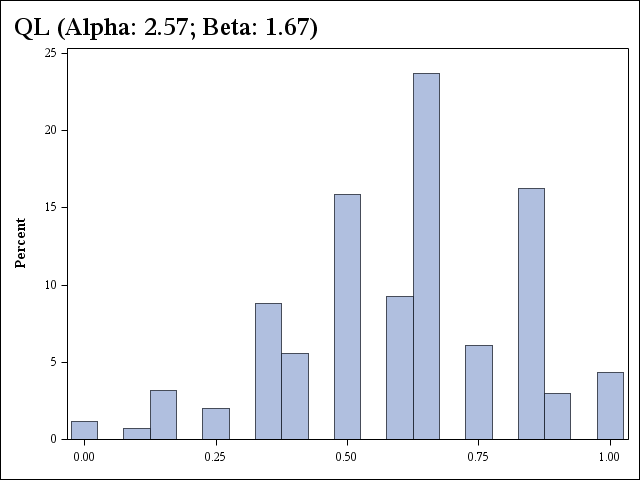 |
| 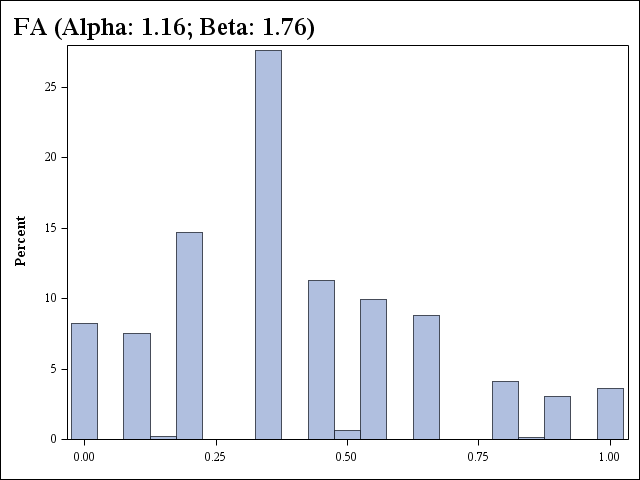 | 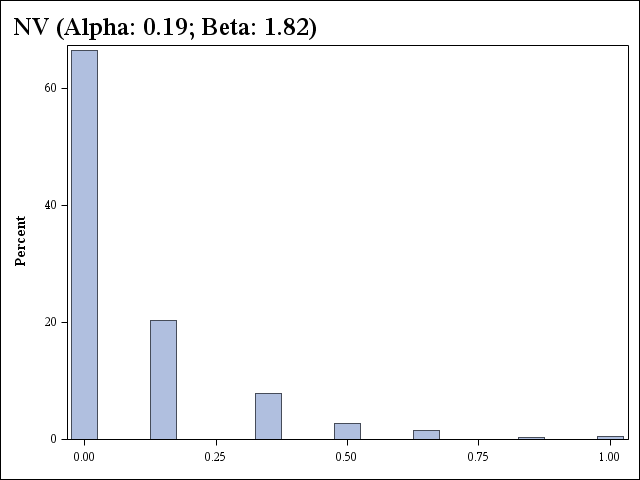 | 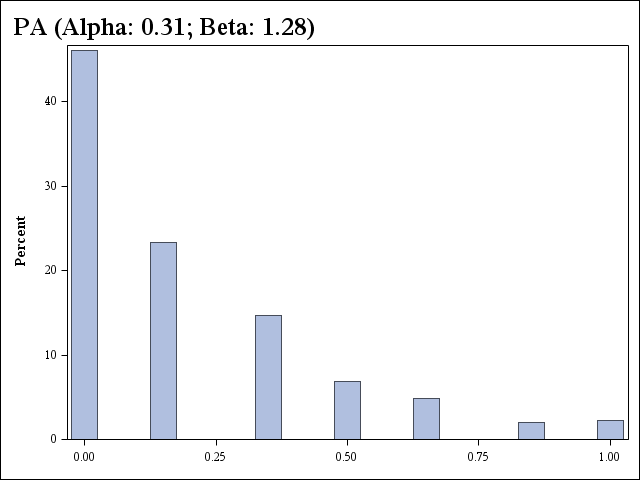 |
| 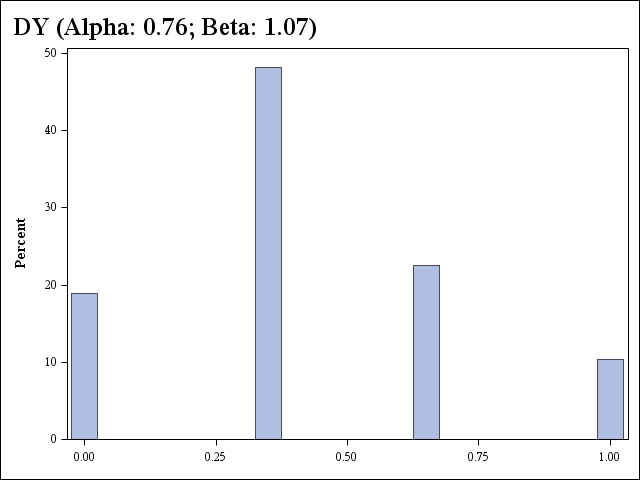 | 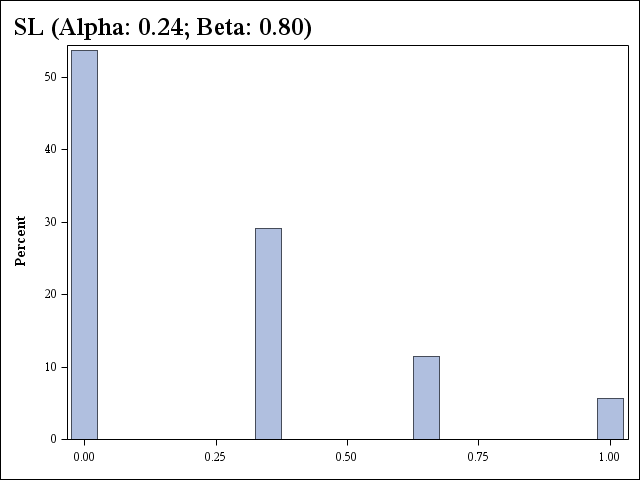 | 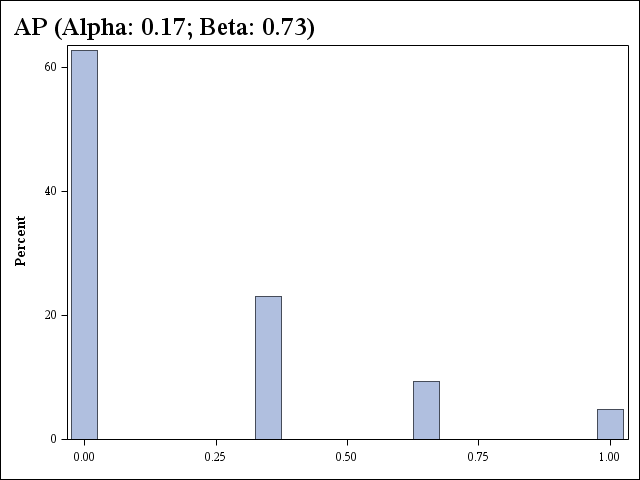 |
| 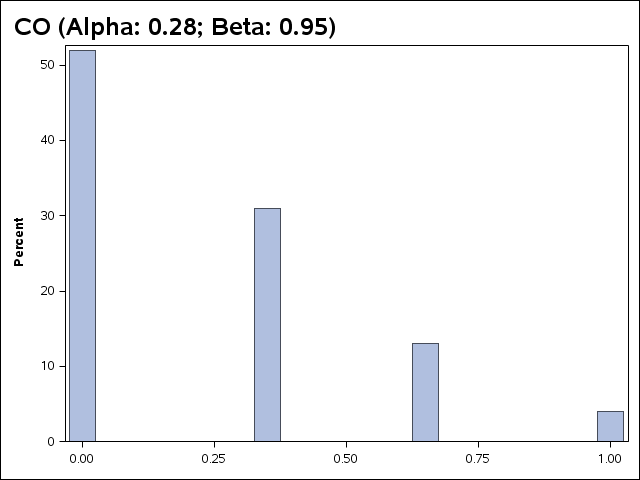 | 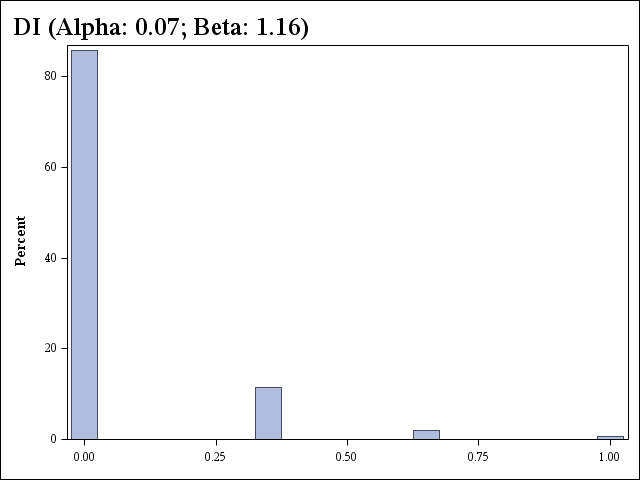 | 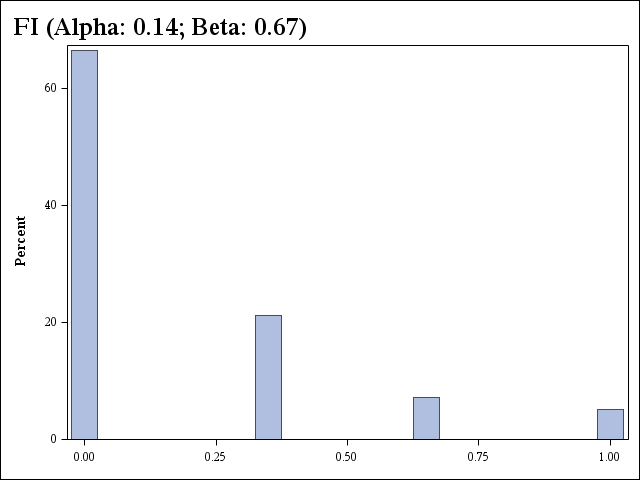 |

**Supplementary Figure 2: Plot of Odds Ratios vs. MDs for all 15 domains (all trials)**

1.
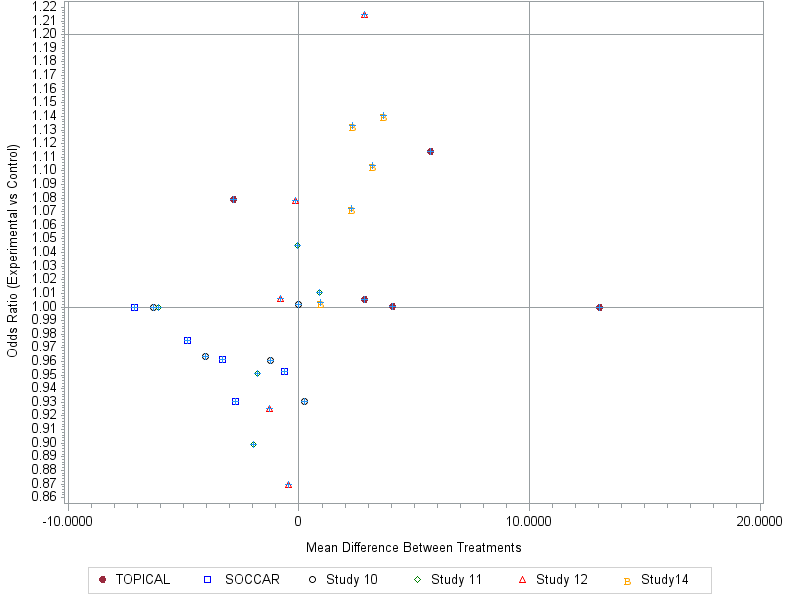

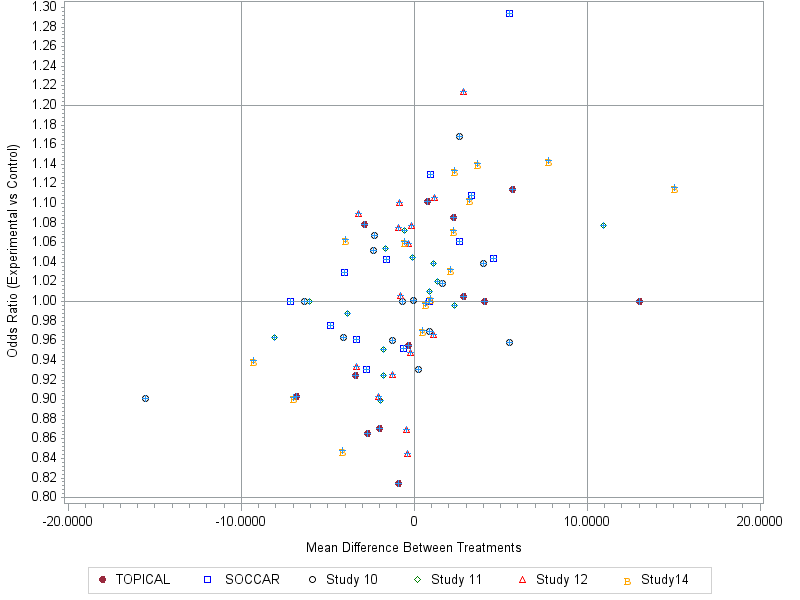
**Overall b) Functional Domain**
2. **Symptom Domain**

**
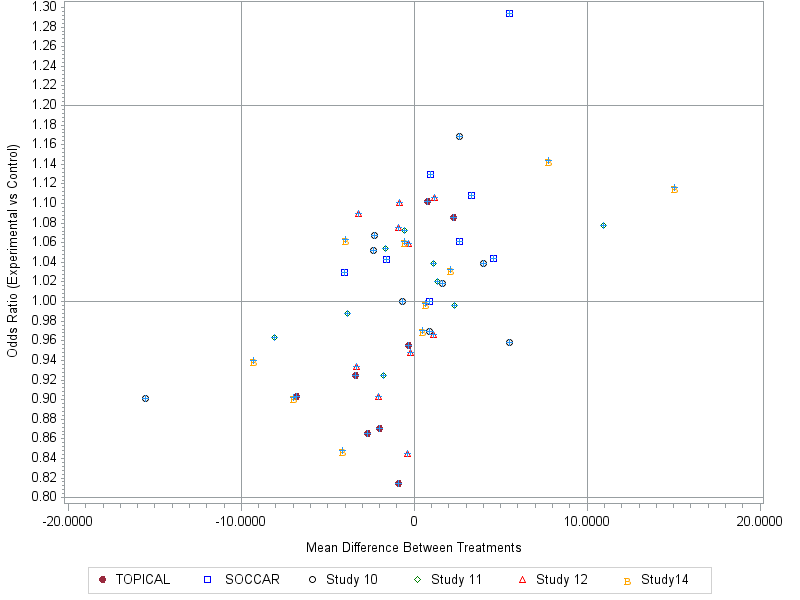
**

Note: Vertical reference lines are +10 points for MDs and zero (no effect line). Horizontal reference lines are 1 (no effect) and 0.8 and 1.20 for ORs.

**Supplementary Figure 3(a): Comparison of erlotinib vs placebo responses for EF in TOPICAL trial**

Example showing MDs not statistically different but ORs statistically significant: Higher proportion of placebo responses for lower scores and higher proportion of erlotinib responses in some categories of emotional function (EF) >0.6 (or 60); distribution is skewed.

**
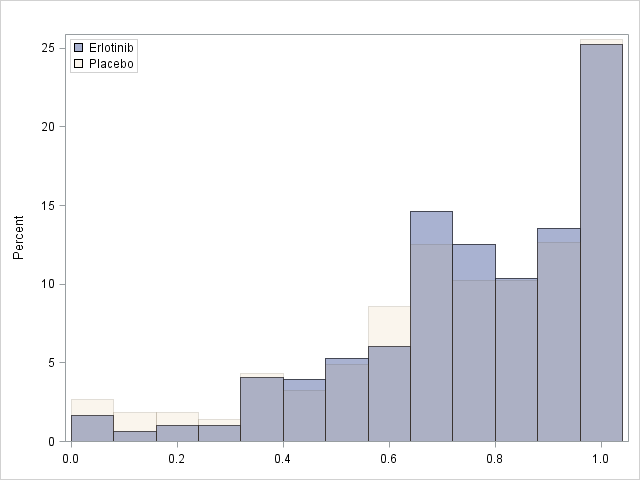
**

**Supplementary Figure 3(b): Comparison of erlotinib vs placebo responses for CO in TOPICAL trial**

**
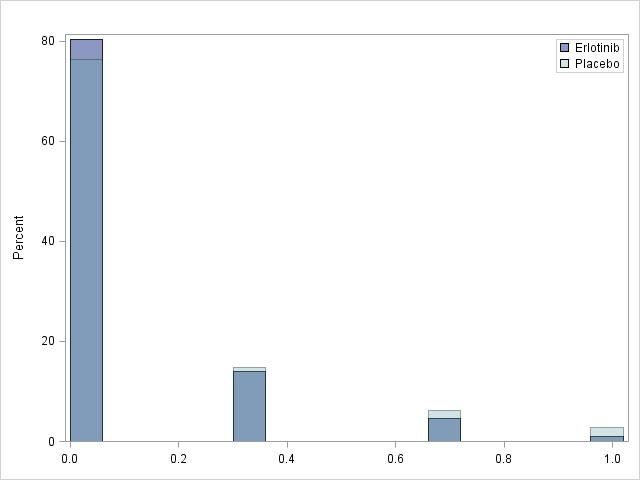
**

Example showing MDs are statistically different but ORs are not statistically significant.

**Supplementary Table 1: summary of QLQ-C30 CRF completion rates at each cycle**

|  |  | |  | |  | |  | |  | |
| --- | --- | --- | --- | --- | --- | --- | --- | --- | --- | --- |
|  | **TOPICAL**  **N=670** | **SOCCAR**  **N=130** | | **Study 10**  **N=241** | | **Study 11**  **N=422** | | **Study 12**  **N=724** | | **Study 14**  **N=722** |
| Baseline | 95% | 96.2% | | 62% | | 91% | | 95% | | 97% |
| Cycle 1 | 73% | 76.2% | | 53% | | 87% | | 81% | | 89% |
| Cycle 2 | 49% | 68.5% | | 43% | | 81% | | 75% | | 79% |
| Cycle 3 | 43% | 69.2% | | 39% | | 74% | | 64% | | 71% |
| Cycle 4 | 33% | 54.6% | | 30% | | 55% | | 51% | | 62% |
|  |  |  | |  | |  | |  | |  |
|  |  |  | |  | |  | |  | |  |

**Supplementary Table 2: Statistical agreement between OR and MD**

|  |  | **Odds Ratio** | | **Total** |
| --- | --- | --- | --- | --- |
|  |  | **p-value <0.05** | **p-value >0.05** |  |
|  | **p-value <0.05** | **14 (67%)** | **18 (26%)** | **32** |
| **MD** | **p-value >0.05** | **7 (33%)** | **51 (74%)** | **58** |
|  |  |  |  |  |
|  | **Total** | **21** | **69** | **90** |

^1^Agreement occurs when the p-value is statistically significant (i.e. <0.05) for both MD and ORs or when the p-value is >0.05 for both

**Supplementary Table 3: Distribution of statistically agreed/disagreed conclusions**

|  |  |  | | |
| --- | --- | --- | --- | --- |
|  | **MD**  **(N=90)** | **OR**  **(N=90)** | **Disagreement^1^**  **(N=7)** | **Disagreement^2^**  **(N=18)** |
| Non SS* results  SS results | 58 (64%)  32 (36%) | 69 (77%)  21 (23%) |  |  |
| Large MD^3^ | 2 (2%)  2 (2%)  22 (24%)  6 ( 7%) | | 0 | 1 |
| Medium MD^4^ |  |  | 0 | 0 |
| Small MD^5^ |  |  | 0 | 13 |
| Trivial MD^6^ |  |  | 7 | 4 |

*SS: Statistically significant ; MD: Mean Difference; OR: Odds Ratio

^1^MD p-value >0.05, but OR p-value <0.05

^2^MD p-value <0.05, but OR p-value >0.05

^3^Large: >15 points in either direction

^4^Medium: >10 and <15 points in either direction

^5^Small: >3 and <10 points in either direction

^6^Trivial: >0 and < 3 points in either direction

**Supplementary Table 4: Distribution of MDs and ORs**

|  |  |  | **Odds Ratios** |  |  |
| --- | --- | --- | --- | --- | --- |
|  | 0.95 -1.0  1.0-1.05 | 0.90-0.95  1.05-1.10 | 0.80-0.90  1.10-1.20 | <0.80  >1.20 | **Total** |
| **Mean Difference** |  |  |  |  |  |
| 0 - 3^1^ | 25 | 21 | 12 | 1 | **59** |
| 3 - 10^2^ | 10 | 10 | 6 | 1 | **27** |
| 10 - 15^3^ | 0 | 1 | 0 | 1 | **2** |
| >15^4^ | 0 | 1 | 1 | 0 | **2** |
| **Total** | **35** | **33** | **19** | **3** | **90** |

^1^Trivial: >0 and < 3 points in either direction

^2^Small: >3 and <10 points in either direction

^3^Medium: >10 and <15 points in either direction

^4^Large: >15 points in either direction

**Supplementary Table 5: Time to Deterioration (>** 5 points) -TOPICAL

| **Treatment** | **Median**  **(Months)** | **QLQ-C30** | **Hazard**  **Ratio** | **p-value** |
| --- | --- | --- | --- | --- |
| Erlotinib | 161 | PF | 1.143 | 0.4452 |
| Placebo | 258 |  |  |  |
| Erlotinib | 147 | EF | 0.829 | 0.2406 |
| Placebo | 100 |  |  |  |
| Erlotinib | 247 | RF | 0.774 | 0.1481 |
| Placebo | 178 |  |  |  |
| Erlotinib | n/c | CF | 1.054 | 0.7960 |
| Placebo | n/c |  |  |  |
| Erlotinib | n/c | SF | 0.712 | 0.0720 |
| Placebo | 398 |  |  |  |
| Erlotinib | 94 | QL | 0.852 | 0.2912 |
| Placebo | 70 |  |  |  |
| Erlotinib | 49 | FA | 1.192 | 0.1630 |
| Placebo | 63 |  |  |  |
| Erlotinib | 90 | NV | 1.191 | 0.2423 |
| Placebo | 113 |  |  |  |
| Erlotinib | 98 | PA | 0.879 | 0.3551 |
| Placebo | 97 |  |  |  |
| Erlotinib | 125 | DY | 0.843 | 0.2547 |
| Placebo | 91 |  |  |  |
| Erlotinib | 147 | SL | 0.948 | 0.7440 |
| Placebo | 140 |  |  |  |
| Erlotinib | 63 | AP | 1.479 | 0.0066 |
| Placebo | 113 |  |  |  |
| Erlotinib | 308 | CO | 0.566 | 0.0006 |
| Placebo | 91 |  |  |  |
| Erlotinib | 46 | DI | 3.219 | <.0001 |
| Placebo | 278 |  |  |  |
| Erlotinib | n/c | FI | 0.700 | 0.1112 |
| Placebo | n/c |  |  |  |

n/c: not calculable due to too few events; Physical Function (PF), Role Function (RF), Emotional Function (EF), Cognitive Function (CF) and social functioning (SF); 9 ‘symptom’ scales: Fatigue (FA), Nausea & Vomiting (NV), Pain (PA), Dyspnoea (DY), Insomnia (IN), Appetite Loss (AL), Constipation (CO), Diarrhoea (DI) and Financial Problems (FI); and a global health status score (QL).
